# A *PageRank*-based heuristic for the minimization of open stacks problem

**DOI:** 10.1371/journal.pone.0203076

**Published:** 2018-08-30

**Authors:** Rafael de Magalhães Dias Frinhani, Marco Antonio Moreira de Carvalho, Nei Yoshihiro Soma

**Affiliations:** 1 Federal University of Itajubá, Mathematics and Computer Sciences Institute, Itajubá, Minas Gerais, 37500-903, Brazil; 2 Federal University of Ouro Preto, Computer Sciences Department, Ouro Preto, Minas Gerais, 35400-000, Brazil; 3 Technological Institute of Aeronautics, Computer Sciences Division, São José dos Campos, São Paulo, 12228-900, Brazil; Mathematical Institute, HUNGARY

## Abstract

The minimization of open stacks problem (MOSP) aims to determine the ideal production sequence to optimize the occupation of physical space in manufacturing settings. Most of current methods for solving the MOSP were not designed to work with large instances, precluding their use in specific cases of similar modeling problems. We therefore propose a *PageRank*-based heuristic to solve large instances modeled in graphs. In computational experiments, both data from the literature and new datasets up to 25 times fold larger in input size than current datasets, totaling 1330 instances, were analyzed to compare the proposed heuristic with state-of-the-art methods. The results showed the competitiveness of the proposed heuristic in terms of quality, as it found optimal solutions in several cases, and in terms of shorter run times compared with the fastest available method. Furthermore, based on specific graph densities, we found that the difference in the value of solutions between methods was small, thus justifying the use of the fastest method. The proposed heuristic is scalable and is more affected by graph density than by size.

## Introduction

The cutting-stock problem occurs in industrial settings in which smaller objects of different sizes and shapes are manufactured to meet customer demands from larger objects of predefined size, such as wood panels, paper or steel rolls and flat glass. The arrangement of smaller objects, i.e., pieces, in larger objects defines a pattern. At each stage of the production process, a pattern is processed, and the resulting pieces are added to specific stacks close to the machine that produced them. However, in this case, physical constraints prevent the allocation of space for the simultaneous accommodation of stacks of all requested pieces. To avoid the risk of damaging products (e.g., glass) and to reduce logistics costs, once a stack is open, it can only be closed and moved to make room when the demand for pieces of the same type has been met.

The minimization of open stacks problem (MOSP) [1] aims to determine the ideal pattern-processing sequence that results in the lowest maximum number of simultaneously open stacks to determine the optimal space allocation in industrial settings. Several problems are similar to the MOSP [2], such as the gate matrix layout problem (GMLP) and programmable logic array folding (PLA Folding). Related problems include the minimization of tool switches problem (MTSP) and minimization of discontinuities problem (MDP), among others [2].

**Fig 1:**
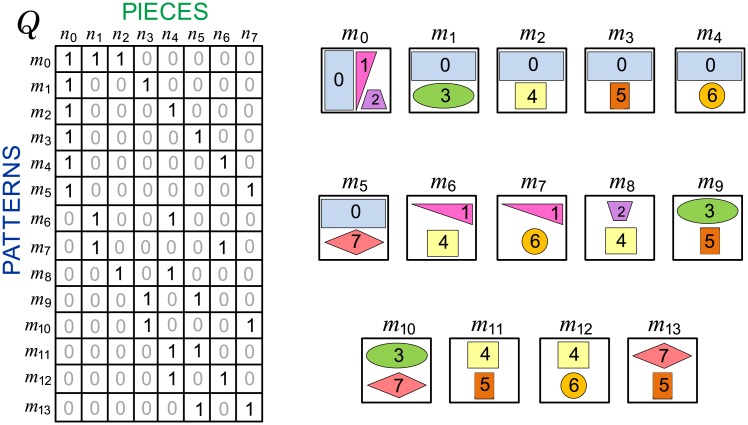
Example of an input matrix of an MOSP instance [3], which represents a set of patterns and their pieces. The instance has 14 patterns *m*_*i*_ with values *i* = {0, …, 13} and 8 pieces *n*_*j*_ with values *j* = {0, …, 7}. In the matrix, the 1s are highlighted for better readability. The patterns and their respective pieces are shown next to the matrix.

The MOSP input data are represented by a binary matrix *Q* with dimensions *m* × *n*, where *m* is the number of patterns and *n* is the number of pieces. Fig 1 shows an example of an input matrix of an MOSP instance. Some data are disregarded in the model, such as the number of pieces of the same type in the composition of a pattern, the maximum stack height and the different processing times of each type of piece.

**Fig 2:**
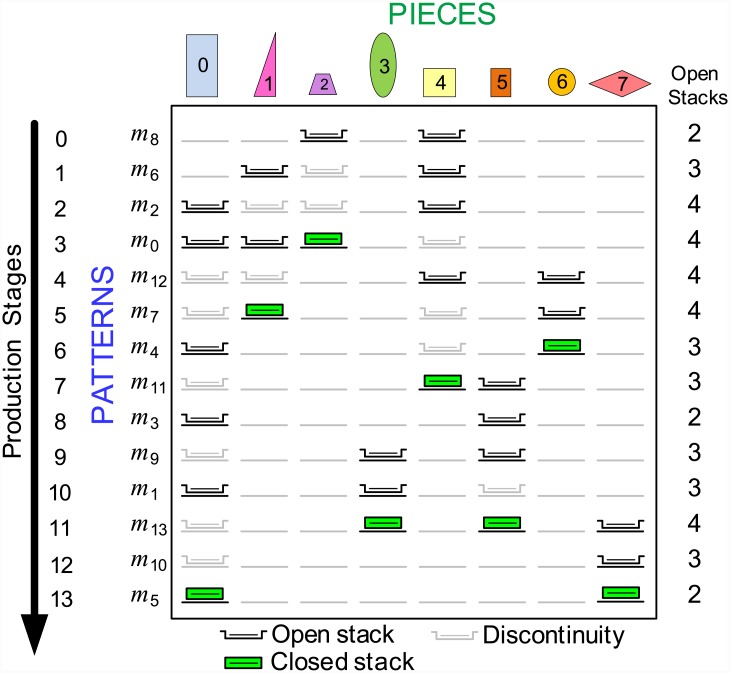
Example of pattern sequencing for the input matrix *Q* of Fig 1. The *MNOS* is 4 for this pattern sequence.

Fig 2 shows an example of a sequence of patterns *π*_*PA*_ for the data input matrix shown in Fig 1. The arrow, indicates the direction of production; that is, *m*_8_ was the first pattern manufactured and *m*_5_ the last. Icons in gray represent a discontinuity, which is an open stack that received no pieces at that production stage but cannot be removed because pieces must still be added to the stack. Black icons represent an open stack that receives pieces at certain stage of production. The green icon represents a stack that received the last piece and, in this case, can be closed and removed in the next stage. The Open Stacks column has the number of open stacks (*NOS*) in each production stage. The maximum number of open stacks (*MNOS*) is 4 for the pattern sequence *π*_*PA*_ = [*m*_8_, *m*_6_, *m*_2_, *m*_0_, *m*_12_, *m*_7_, *m*_4_, *m*_11_, *m*_3_, *m*_9_, *m*_1_, *m*_13_, *m*_10_, *m*_5_]. For example, the consecutive 1s property [4] is used to calculate the *NOS* at each production stage. $Q_{\pi_{PA}}^{1}$ is the permutation matrix resulting from *Q* according to the sequence of patterns *π*_*PA*_ such that, in any column $Q_{\pi_{PA}}^{1}$, each cell between 1s also has the value 1. The *NOS* of a production stage may be calculated by adding the 1s of the corresponding row in the matrix $Q_{\pi_{PA}}^{1}$. *MNOS* is given by the largest *NOS*. An optimal solution for the MOSP is that in which the maximum number of 1s in the same row of matrix $Q_{\pi_{PA}}^{1}$ is the lowest possible.

To describe the problem more formally, the mathematical formulation of the MOSP by Yanasse & Pinto [5], transcribed below, determines the order in which the stacks of pieces are closed. *C* is the maximal number of open stacks, *e* is an auxiliary 1 × *n* vector of 1s, *t* is the stage immediately after completing the stack of the *t*^*th*^ piece, and *n* is the total number of pieces. *W*_*t*_ is a binary *n* × 1 vector that provides the pieces whose stacks are already open or closed at stage *t*, for *t* = 0, …, *n* − 1. *x*_*jt*_ is 1 if the stack of piece *j* is the *t*^*th*^ stack closed and 0 otherwise. *S*_*k*_ is a binary *n* × 1 vector that indicates the other pieces that occur in the same pattern as piece *k*; that is, *S*_*jk*_ is 1 if pieces *j* and *k* are in the same pattern. Finally, *K* is a sufficiently large constant (*K* ≥ *n*).

$$\begin{array}{c} Minimize\;C \end{array}$$ (1)

$$\begin{array}{c} Subject\;to\\ \begin{array}{c} eW_{t}\leq C+t\;-\;1,\;t=0,\ldots ,n-1 \end{array} \end{array}$$ (2)

$$\begin{array}{c} \sum_{j=0}^{n-1}\sum_{k=0}^{t}x_{jk}S_{j}\leq KW_{t},\;t=0,\ldots ,n-1 \end{array}$$ (3)

$$\begin{array}{c} \sum_{t=0}^{n}x_{jt}=1,\;j=0,\ldots ,n-1 \end{array}$$ (4)

$$\begin{array}{c} \sum_{j=0}^{n-1}x_{jt}=1,\;t=0,\ldots ,n-1 \end{array}$$ (5)

$$\begin{array}{c} x_{jt}\in \left\{0,1\right\},\;j=0,\ldots ,n-1;\;t=0,\ldots ,n-1 \end{array}$$ (6)

$$\begin{array}{c} W_{t}\;is\;a\;binary\;array. \end{array}$$ (7)

To close a stack corresponding to a piece *j*, all patterns containing this piece must be processed. After process all patterns that complete one stack of pieces, at least one stack is closed. The resulting *NOS* is equal to the number of open stacks plus the number of closed stacks. Thus, all resulting stacks after attend the demand of each piece are always the stacks that were present in the previous closing of some stack of pieces plus the new stacks opened to close the current stack of pieces. The objective of model (1) is to minimize the maximal number of open stacks *C*. After the closure of the *t*^*th*^ stack, *t* stacks are closed. During the closing of the *t*^*th*^ stack, a total of *C* stacks are open plus the number of stacks already closed in stage *t* − 1. Constraint (2) relates the total number of open and closed stacks during the closing of a stack and should be less than or equal to the *MNOS* plus the stacks closed up to stage *t* − 1. Constraint (3) indicate that if a stack of determined piece *k* is closed, every piece *j* that appears with piece *k* in some pattern will also form a stack. Constraints (4) and (5) indicate that each stack will be closed in some order. Finally, constraints (6) and (7) correspond to the integrality of decision variables.

**Fig 3:**
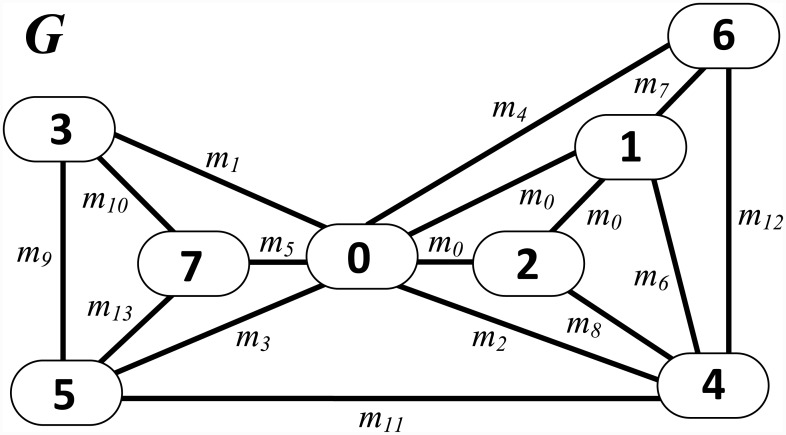
MOSP graph modeling for the input matrix is shown in Fig 1, adapted from [3]. The pattern *m*_0_ is a 3-node clique, and all other patterns are represented by 2-node clique.

To solve the MOSP, methods using *Q* column or row permutation have been proposed [4, 6, 7], whereas other authors have adopted graph modeling [3, 8, 9]. In this case, sequencing is associated with a search problem. In the MOSP graph [10], the nodes represent pieces, and the edges connect pieces with common patterns. It should be noted that in an MOSP graph, all pieces of the same pattern are connected to each other, constituting a clique (i.e., a complete subgraph), disregarding loops (i.e., edges with the origin and destination at the same node) and parallel edges (i.e., sets of edges with the same origin and destination nodes) from the model. Edges are unweighted (i.e., have no values) and have no direction defined (i.e., are bidirectional or undirected). The MOSP graph *G*, shown in Fig 3, was constructed from the input matrix *Q* shown in Fig 1.

The *First Constraint Modelling Challenge* [7] increased the visibility of MOSP in the scientific community. Various methods have been proposed to solve this problem, including heuristics [1, 3, 8, 9, 11], metaheuristics [12–14], exact [6, 15–17] and hybrid [18, 19] methods. Among state-of-the-art methods, we highlight the minimal-cost node heuristic (*MCNh*) [3], the fastest method; the heuristic *HBF_2r_* [9] and the metaheuristic *BRKGA* [14], which have the best relationship between solution quality and computing time; and the exact method developed by *Chu & Stuckey* [17]. The Materials & Methods section includes a brief description of the methods used in the experiments. In addition to sequencing methods, preprocessing operations were also proposed [4] being useful for reducing the dimension of instances and, consequently, the runtime.

The MOSP is NP-hard [2], and because P≠NP, unless proven otherwise, the processing time and memory consumption will increase or the solution quality will decrease with increasing dimension of the problem. However, solving large instances is necessary in specific similar problems, such as GMLP and PLA folding, associated with very-large-scale integration (VLSI) circuit projects [20]. The objective is to find an arrangement of logic gates, consisting of transistors, that minimizes the maximum number of necessary paths to interconnect them, thereby enabling the construction of more compact and less expensive printed circuits. In a more recent scenario observed for the main automotive manufacturers of the Brazilian market [21], MOSP-based modeling was used to optimize the use of space in local stock, with instances larger than 1000 items in a manufacturing cell. The numerous vehicle configuration possibilities and the wide variety of pieces increase the complexity of inventory logistics and the amount of space necessary for the local stock. The objective is to find the ideal vehicle assembly sequence that minimizes the variety of pieces and the occupied space.

In general, the sequencing methods available in the literature were not designed to solve large instances, considering the practical applications hitherto. The exact method of *Chu & Stuckey* can solve instances then classified as difficult [9] within 13 hours of processing for a single 200 × 200 instance, which was the largest dimension analyzed. Alternatives to exact methods, *HBF_2r_* and *BRKGA*, obtain good solutions faster. These methods use local search and assess multiple calculations of the objective function, which are time consuming for large instances because the structure of the problem requires similarly large matrices for its representation. To obtain a good runtime-to-solution-quality ratio, in addition to developing efficient search and enumeration strategies, methods that provide good-quality initial solutions in the shortest runtime possible must be developed to reduce the number of improvements. In this context, ad hoc heuristics stand out for their simple sequencing strategy, followed by a single calculation of the objective function. Examples include the methods *Yuen3* [1, 8], *Ashikaga & Soma* (AS) [11] and *MCNh* [3]. *Ashikaga & Soma* considered solving 1000 × 1000 MOSP instances in their experiments. To our knowledge, this is the only study solving large MOSP instances. Although *MCNh* is the most computationally expensive of the three methods, it provides the highest-quality solutions. Details on the performance of these methods are available in the Supporting Information S1 Table—Detailed Results of Experiments.

To compute large instances of graph-modeled data, the use of alternative methods of analysis may improve their understanding and the development of new sequencing strategies. Network science is an academic field based on the application of theories and methods from several areas, such as graphs, statistics, physics, computing and sociology [22], to the study of complex networks characterized by large dimensions and non-trivial iteration patterns. A complex network is modeled as a graph whose nodes and edges represent its elements and interactions, respectively. The measures used to analyze complex networks improve the understanding of its structure (e.g., dimension, density and connectivity) [23] and interactions (e.g., clustering coefficient and centrality) [24, 25]. In particular, centrality measures the importance of each node to the entire network structure [26]. Several methods can be used for this calculation [27–30], among which we highlight *PageRank* [31, 32], which was developed to measure the global importance of each webpage returned in a query. Due to its simplicity and fast computation, *PageRank* has been applied to similar situations in other fields, such as chemistry, biology, systems engineering, social networks and referral systems [26, 33–35]. Its operation will be detailed in the Materials and Methods section.

Considering the above, the objective of this study is to propose a method to solve large instances of the MOSP. The sequencing strategy adopted is based on the traversal of the nodes in an MOSP graph. The order in which the nodes are visited defines the sequence of pieces. Based on a measure and on a search criterion, the sequencing of specific nodes is prioritized. To identify the priority pieces in the sequencing of a large graph, we compare the MOSP with *PageRank* in the Web page ranking problem. In this case, the pieces replace the webpages, and centrality indicates their importance in relation to the other pieces. As a first contribution, we highlight the development of a sequencing heuristic termed *PieceRank*, which uses *PageRank* to assess the centrality of pieces in an MOSP graph. The short runtime of the *PageRank*, even for graphs with millions of nodes, was an additional motivation for its use. The size of the instances in the application scenario analyzed—industrial cutting-stock problems—is significantly smaller than that in the Web scenario. This condition reduces the effort of *PageRank* and helps quickly calculate the centrality value of all pieces.

*PieceRank* uses a simple sequencing strategy based on a greedy search on the MOSP graph, whose choice of the next node does not depend on the solution of the subproblems (e.g., dynamic programming). As a criterion of choice, the node with the lowest centrality value is prioritized in the path. From the first node, its adjacent nodes are traversed until all have been visited, in a procedure similar to a breadth-first search. In addition, adopting a node-based strategy has advantages over methods based on the edge traversal of the graph (e.g., *MCNh*). In the MOSP graph, the representation of the patterns in a clique leads to a significantly higher number of edges than of nodes, considering that (*n*^2^ − *n*)/2 edges will exist for each clique of *n* nodes. The *PageRank* performance and the adoption of a simple sequencing strategy help to reduce the *PieceRank* runtime, which, in some cases, is 20 times shorter than that of the fastest state-of-the-art method when solving large instances. Details on the operation of *PieceRank* are provided in the Materials and Methods section.

This study also innovates by adopting centrality as an alternative to commonly used measures based on node degree or on the search for maximum cliques. In contrast to degree, which analyzes each relationship equally, in *PageRank*, relationships with more influential nodes are valued more than those with less influential nodes. This feature provides a broad view of the importance of the node in the graph, in contrast to the narrow view associated with degree-based strategies. The more detailed description of the importance of the node also enables the identification of more promising regions in the graph to start the sequencing, that is, the regions whose nodes are best related to meeting the defined search criteria. Additionally, to limit the premature opening of new stacks, the heuristic prioritizes the sequencing of pieces with the lowest number of relationships, which are the pieces with the lowest *PageRank* values. The results show the competitiveness of *PieceRank*, which is able to find optimal or good-quality solutions in several cases, compared with state-of-the-art methods.

An additional contribution of this study is the analysis of MOSP graphs based on density and not merely dimension. MOSP graphs that has specific structures (e.g., tree, 1-tree, *k*-regular or complete graph), are easily identified and have a trivial solution [4]. To best assess performance in other cases, we sought to identify a possible convergence between methods in the value of solutions on specific densities. Proposing a classification for MOSP graphs is not the purpose of this study, as we understand the need for more comprehensive analyses that include, in addition to graph features, the use of classification methods. However, the results showed that, above specific densities, the difference in the value of solutions between methods is very small, justifying the adoption of the fastest method. We also observed that the runtimes of *MCNh*, *HBF_2r_*, and *Chu & Stuckey* are more strongly affected by an increase in problem dimension, whereas *PieceRank* is more scalable because this method is more sensitive to the increase in graph density.

The remainder of this article is organized as follows. The Materials and Methods section describes the operation of *PageRank* and of the proposed method, in addition to briefly describing the methods used in the experiments. The Results section details the experimental design used and the findings. Lastly, the Conclusion and Future Studies section summarizes the main contributions of the study and recommendations for future works.

## Materials and methods

This section begins with an operational description of *PageRank*, which is the centrality-based method for the sequencing approach proposed. Then, the proposed approach, termed *PieceRank*, is described, and its execution is exemplified. Lastly, the methods used in the experiments are briefly described.

### PageRank

**Fig 4:**
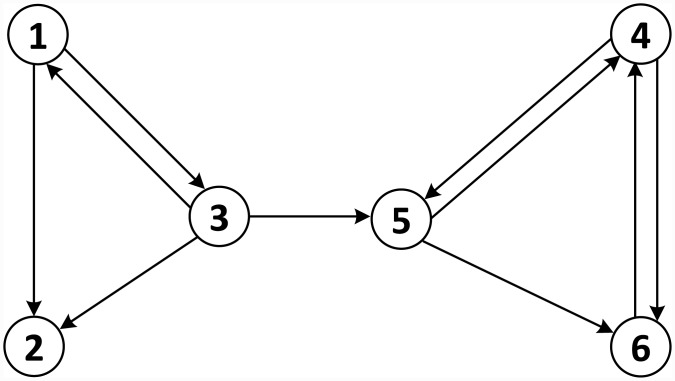
Directed graph representing a Web with six pages [37].

Suppose a small Web with six pages and respective links, as shown in Fig 4. Intuitively, *PageRank* works as a “surfer” that surfs the Web via random choices of links available from the initial page visited [31]. At specific times, the surfer may be bored with the available link options and choose to go to a new page by typing a new address in the browser. The probability of the random surfer visiting a page through a link is the *PageRank* value, and the probability of breaking the chain of links and visiting a new page by typing in the address is termed the *damping factor*.

Formally, *G* = *(V, E)* is a directed graph representing the Web, with a set of nodes *V* representing the pages and an set of edges *E* representing links between pages, where *E* is a subset of *V* × *V*. An edge incident to a node *p*_*i*_ is termed an *inlink* if it originates at a node *p*_*j*_ and links to *p*_*i*_ and is termed an *outlink* if it originates at a node *p*_*i*_ and links to *p*_*j*_. As example, in the graph of Fig 4, the node 3 has one *inlink* and two *outlinks*. A node *p* is considered a *hub* if it has more *outlinks* than *inlinks* and is considered an *authority* if it has more *inlinks* than *outlinks*. The more *inlinks* a page has, the more important it will be.

As defined by Page [31], the *PageRank* value of page *p*_*j*_, termed *r*(*p*_*j*_), is the sum of the *PageRank* values of all pages linking to *p*_*j*_, according to Eq 8:

$$\begin{array}{c} r_{z+1}\left(p_{j}\right)=\sum_{p_{k}\in B_{p_{j}}}\frac{r_{z}\left(p_{k}\right)}{|p_{k}|} \end{array}$$ (8)

in which $B_{p_{j}}$ is the set of pages linking to *p*_*j*_ and |*p*_*j*_| is the total number of *outlinks* of page *p*_*j*_. The *PageRank* value is successively calculated in an iterative process in which the previous values of *r*(*p*_*j*_) are replaced at each iteration *z* until the convergence of the method. Because the *PageRank* values are unknown in the beginning, before the first iteration, all pages are assumed to have the same *PageRank*, with value is given by 1/|*p*|, where |*p*| is the total number of pages. Thus, the process begins with *r*_0_(*p*_*j*_) = 1/|*p*| for all pages *p*_*j*_.

Table 1 outlines the change in the *PageRank* value in the first three iterations. Initially, in the Iteration 0 column, all nodes are given the same *PageRank* value. From Iteration 1, the *PageRank* of each node is calculated using Eq 8. As an example calculation, in the Iteration 1 column, the *PageRank* value of page *p*_2_ is given by $r_{1}\left(p_{2}\right)=\frac{1/6}{2}+\frac{1/6}{3}=5/36$, considering that the pages linking to *p*_2_ are *p*_1_ and *p*_3_, whose *PageRank* is 1/6, and that the numbers of *outlinks* are 2 and 3, respectively.

**Table 1:** Example of the initial *PageRank* iterations according to Eq 8 for the graph of Fig 4, adapted from Langville [37].

Iteration 0	Iteration 1	Iteration 2	Rank at Iter 2
*r*_0_(*p*_1_) = 1/6	*r*_1_(*p*_1_) = 1/18	*r*_2_(*p*_1_) = 1/36	5
*r*_0_(*p*_2_) = 1/6	*r*_1_(*p*_2_) = 5/36	*r*_2_(*p*_2_) = 1/18	4
*r*_0_(*p*_3_) = 1/6	*r*_1_(*p*_3_) = 1/12	*r*_2_(*p*_3_) = 1/36	5
*r*_0_(*p*_4_) = 1/6	*r*_1_(*p*_4_) = 1/4	*r*_2_(*p*_4_) = 17/72	1
*r*_0_(*p*_5_) = 1/6	*r*_1_(*p*_5_) = 5/36	*r*_2_(*p*_5_) = 11/72	3
*r*_0_(*p*_6_) = 1/6	*r*_1_(*p*_6_) = 1/6	*r*_2_(*p*_6_) = 14/72	2

Although initially developed for directed graphs, *PageRank* can also be applied to undirected graphs [36]. Several problems are naturally modeled as undirected graphs, for example, social networks, relationships between genes, protein-protein interactions and in neuroscience [26]. Undirected graph modeling primarily results in a symmetric adjacency matrix. By comparison, Gabor [36] considered that the *PageRank* value is proportional to the degree of nodes in an undirected graph and therefore would not facilitate the selection of more important or less important network nodes compared with a simple degree count. However, more recent results have shown that the *PageRank* value in an undirected graph is proportional to the distribution of degrees only in regular graphs [38, 39]. Mihalcea [40] highlights that undirected, sparse graphs with the number of edges proportional to the number of nodes tend to have more gradual convergence curves obtained after a few iterations and that the curves of directed and undirected graphs virtually overlap.

### PieceRank heuristic for the MOSP

The heuristic *PieceRank* uses *PageRank*-based centrality as a measure to solve the MOSP. Thus, similar to the methods *MCNh* and *HBF_2r_*, *PieceRank* uses MOSP graph modeling. The intuition behind the proposed heuristic is the use of *PageRank* to obtain a vector that stores the centrality value of each piece, which can be regarded as its importance in relation to all others. More specifically, the centrality of the piece represents the probability that this piece is related to other pieces of the problem. A piece with a high *PageRank* value has relationships with several other pieces. Similar to *MCNh* and to the breadth-first search of *HBF_2r_*, *PieceRank* obtains a sequence of pieces, and the corresponding pattern sequence is obtained according to the procedure proposed by Yanasse [6]. This procedure is further explained in the next section.

The principle of operation of *PieceRank* is to prioritize the sequencing of pieces with the fewest relationships with others, thereby limiting the premature opening of stacks in the production stage in which the pattern is sequenced. For both *MCNh* and *HBF_2r_*, the relationship in question is given by the node degree. *MCNh* is based on the analysis of edges, which receive a weight calculated as the sum of the degrees of the nodes they connect, and *HBF_2r_* is based on the degree of the node itself. The objective of *MCNh* is to prioritize the sequencing of the node whose edge has the lowest weight; the objective of *HBF_2r_* is to prioritize the sequencing of the node with the lowest degree. *PieceRank* adopts the same strategy but differs by considering the value of node centrality and not its degree directly. A characteristic of eigenvector-based centrality methods, such as *PageRank*, is that a node is important if it has important neighbors. This concept applied to an MOSP graph provides a broader view of the importance of each piece by analyzing data on neighboring pieces. This strategy prioritizes the sequencing of a less important piece if it is located in a region whose pieces are also less important.

Algorithm 1 contains the details of the *PieceRank* heuristic for piece sequencing, which has the MOSP graph *G* as input and returns the sequence of pieces *π*_*PI*_. In line 1, the list *π*_*PI*_ corresponds to the sequence of pieces found by the heuristic, and the *CP* list corresponds to the candidate pieces already added to *π*_*PI*_ but whose adjacent pieces have not yet been all analyzed. In line 2, the vector *vetCent*, which stores the *PageRank* value of each *G* piece, is obtained. This vector will be used as a reference to create the *rankPieces* list in line 3. The *rankPieces* list includes the indices of the pieces in non-decreasing order of the value obtained by summing the *PageRank* values of the piece and of its adjacent pieces, which we will term the cumulated *PageRank*.

**Algorithm 1:** *PieceRank*

**Data:** *G*

**1** *π*_*PI*_ = [ ]; *CP* = [ ];

**2** *vetCent* ← getPageRank(*G*);

**3** *rankPieces* ← buildRankOfPieces *(vetCent)*;

**4** *piece*_*ref* ←_ *rankPieces*[0];

**5** *π*_*PI*_, *CP* ← *piece*_*ref*_;

**6** *rankPieces* ← *rankPieces* − *piece*_*ref*_;

**7** *adjtsPiece*_*ref*_ ← getAdjacents(*piece*_*ref*_);

**8** *rankPieces*.updatePagerank(*adjtsPiece*);

**9** **while** *rankPieces* ≠ ∅ **do**

**10**  *adjtsPiece*_*ref* ←_ sortPiecesByPagerank(*adjtsPiece*_*ref*_);

**11**  **for** each adjtPiece ∈ *adjtsPiece*_*ref*_ **do**

**12**   *π*_*PI*_, *CP* ← *adjtPiece*;

**13**   *rankPieces* ← *rankPieces − adjtPiece*;

**14**   *adjtsAdjtPiece* ← getAdjacents(*adjtPiece*);

**15**   *rankPieces*.updatePagerank(*adjtsAdjtPiece*);

**16**  *CP* ← *CP* − *piece*_*ref*_;

**17**  *CP* ← sortPiecesByPagerank(*CP*);

**18**  *piece*_*ref*_ ← *CP*[0];

**19**  *adjtsPiece*_*ref* ←_ getAdjacents(*piece*_*ref*_) − *π*_*PI*_;

**20** **return** *π*_*PI*_

In line 4, the piece with the lowest value is defined as the reference piece, *piece*_*ref*_, which is the piece whose adjacent pieces will be analyzed for addition to *π*_*PI*_. In line 5, *piece*_*ref*_ is added to the sequence *π*_*PI*_ and to the *CP* list because its adjacent pieces have not been analyzed yet. Specifically, in this line, *piece*_*ref*_ is the first piece to be added to *π*_*PI*_. The piece is then removed from *rankPieces* in line 6. In line 7, a list of the indices of pieces adjacent to *piece*_*ref*_ is obtained. In line 8, the cumulated *PageRank* of each piece adjacent to *piece*_*ref*_ is decremented from the *PageRank* value of *piece*_*ref*_. The justification for this decrease is that because the piece has already been added to *π*_*PI*_, it will no longer affect the value of the cumulated *PageRank* of its adjacent pieces in the following sequencing. *PageRank* is updated only in nodes adjacent to *piece*_*ref*_.

Line 9 controls the iterative procedure that is executed until *rankPieces* is empty. In line 10, the pieces adjacent to *piece*_*ref*_ are sorted in non-decreasing order of cumulated *PageRank*. In line 11, each adjacent piece, termed *adjtPiece*, belonging to *adjtsPiece*_*ref*_ is added to the sequence *π*_*PI*_ and to the *CP* list (line 12). The piece is removed from *rankPieces* in line 13. In line 14, a list of pieces adjacent to the piece *adjtPiece*, termed *adjtsAdjtPiece*, is obtained. The respective cumulated *PageRank* of these adjacent pieces will be decremented from the *PageRank* of *adjtPiece* in line 15.

**Fig 5:**
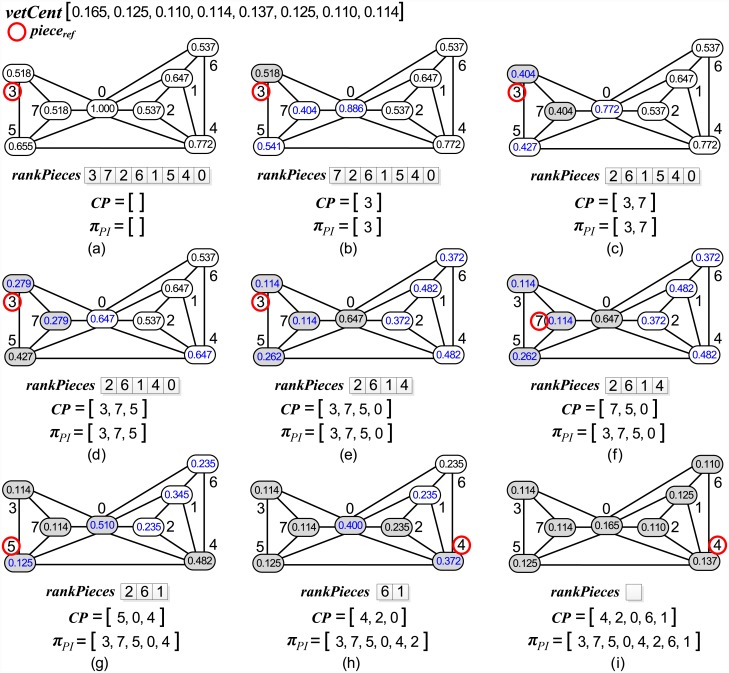
*PieceRank* applied to MOSP graph *G* resulting from input matrix *Q* shown in Fig 1.

Because all its adjacent pieces have already been added, *piece*_*ref*_ is removed from *CP* in line 16. In line 17, *CP* is sorted in non-decreasing order of cumulated *PageRank*. This order differentiates the sequencing method from a conventional breadth-first search. During the breadth-first search, the order of exploration of nodes is determined by a priority queue, which contains nodes whose neighborhoods were not yet explored. The sequencing in this case, follows the order that the nodes were added to the priority queue. By contrast, the proposed heuristic defines the next node for analysis as the node with the lowest cumulative *PageRank*. In line 18, the piece with the lowest cumulated *PageRank* is defined as the next *piece*_*ref*_. In line 19, a list of the indices of the pieces adjacent to *piece*_*ref*_ is obtained, disregarding the pieces that already belong to *π*_*PI*_. The algorithm adopts the same procedures mentioned above until the stop condition is met. The final sequence of pieces *π*_*PI*_ is returned by the method in line 20. Fig 5 illustrates how *PieceRank* operates in sequencing pieces.

The vector *vetCent* at the top of the figure contains the *PageRank* value of each piece. The reference piece is highlighted with a red circle, and a gray node indicates that the piece has already been added to the *π*_*PI*_ sequence. The *rankPieces* list below the graph contains the nodes sorted in non-decreasing order of cumulated *PageRank*. The *CP* list stores the pieces that are candidates for analysis of their adjacent pieces. The procedure starts in graph in (a), where the reference piece of the sequencing is defined as the piece with the lowest cumulated *PageRank*. Because pieces 3 and 7 have the same value, the piece with the lowest index is chosen. In (b), the chosen piece is removed from *rankPieces* and added to *π*_*PI*_ and *CP* lists. The cumulated *PageRank* value of each piece adjacent to 3, highlighted in blue, is decremented from the *PageRank* value of the piece added. In (c), the sequencing of pieces adjacent to *piece*_*ref*_ begins, prioritizing the piece of index 7 because it has the lowest cumulated *PageRank*. After piece 7 is added to *π*_*PI*_, the cumulated *PageRank* of its adjacent pieces is decremented from its *PageRank* value. The procedure is repeated in (d) and (e), sequencing the pieces 5 and 0 and then updating the cumulated *PageRanks* of the respective adjacent pieces. In (f), the piece of index 3 is removed from the *CP* because all adjacent pieces were added to the sequence. *CP* is sorted according to the cumulated *PageRank*, choosing piece 7 as next reference piece. In this stage, the sequence *π*_*PI*_ remains unchanged because all pieces adjacent to 7 have already been added. The method continues in (g) with piece 5 as reference, and the piece of index 4 is the only adjacent piece that has not been added to *π*_*PI*_ yet. When added to the sequence, the cumulated *PageRank* of its adjacent pieces is decremented. Because all pieces adjacent to piece 5 have been added, in (h), piece 4 is chosen in *CP* as the reference piece, and piece 2 is the adjacent piece added to *π*_*PI*_. The graph in (i) contains the final result from the sequencing, which is reached when no pieces remain in *rankPieces* for sequencing. Lastly, the value in each node is the *PageRank* value of the piece. After running *PieceRank*, the sequence of *π*_*PA*_ patterns must be obtained from the sequence of *π*_*PI*_ pieces, as proposed by Yanasse [6].

### Methods used in the experiments to solve the MOSP

#### *MCNh* [3]

The minimal-cost node heuristic (*MCNh*) is based on the traversal of edges in an MOSP graph, using the lowest number of sequencing edges as the criterion to close a stack. The degrees of visited nodes decrease 1 unit for each visit to one of the edges, and the value of zero indicates that all of the adjacent nodes were visited. The order in which the edges are traversed dictates the sequencing order of the pieces. To obtain the sequence of patterns, a sequence of pieces is traversed from the last to the first piece, which are replaced one by one by the respective patterns and sorted in decreasing index order, as proposed by Yanasse [6].

#### *Chu & Stuckey* [17]

The method of *Chu & Stuckey* is an exact method that expands the method proposed by *La Banda & Stuckey* [16] by combining a history of solutions considered poor, because they prevent an optimal solution from being reached, with the *branch-and-bound* method, which adopts an enumerative strategy to choose the next stack of pieces to be closed. The algorithm relies on dominance analysis of subproblems resulting from the search performed in the tree generated by *branch-and-bound*, thus allowing it to be performed only in patterns requested by the next stack to be closed. The method uses upper bound to guide partial solutions and to reduce the analysis time.

#### *HBF_2r_* [9]

Its operation is divided into three stages: breadth-first search (BFS), pattern sequencing and solution refinement. In the BFS stage, a breadth-first search is performed starting at the lowest-degree node, whose adjacent nodes are visited, prioritizing those of lowest degree. The order in which the nodes are visited determines the sequence of pieces. The pattern sequence is obtained according to the procedure proposed by Yanasse [6]. In the solution refinement stage, two pattern sequencing rules, which anticipate or postpone the closure of stacks, are applied to find the best-quality solutions.

## Results

In this section, we present the experimental design adopted and describe the methods used for comparison, the development environment and the experiments. Subsequently, we present the results.

### Experimental design

The experiments aimed to analyze the application of the *PieceRank* heuristic without using preprocessing or local search operations to solve the graph-modeled MOSP. The analysis compared the proposed method with the sequencing methods *MCNh*, *HBF_2r_* and *Chu & Stuckey*. The results for the variables runtime (milliseconds), solution quality (*MNOS*), and percentage gap between the value of the solution obtained using the method and the optimal value, denoted %gap, calculated as 100 × *(method solution − optimal solution)* / *optimal solution*, were analyzed. In the absence of the optimal value, the best known value will be used as the reference value in the calculations.

The development and the experiments were performed by using an Intel Core i5-2400 processor, 3.1 GHz × 4, 4GB RAM, with a Linux Ubuntu 16.04.1 LTS 64-bit operating system. The *PieceRank* method was implemented in Python 3.5.1 MSC v.1900 64 bit. The iGraph 0.7.1.post6 package [24, 41], which is a widely used collection of network analysis tools, was used to build the graphs and to run the *PageRank* algorithm, whose parameters were defined considering an undirected and unweighted graph, with a 0.35 *damping factor* and prpack implementation. The other parameters were set as standard. The *damping factor* was assessed empirically and the value considered was one that contributed to the best results in terms of *MNOS*. Details on the *damping factor* are described in the Materials and Methods section.

The datasets used in the experiments are commonly adopted in the literature. The characteristics of these datasets are described below. The GP, Miller, NWRS, Shaw and SP datasets were created for the *First Constraint Modelling Challenge* [7].

- **GP** [7]. Dataset with eight instances, namely, four 50×50 and four 100×100 instances, generated using three methods (data not reported by the authors). This dataset had the highest density of all datasets used.
- **Miller** [7]. A single 40×20 instance, without any description of its generation.
- **NWRS** [7]. Eight small and medium instances generated by the methods used for the GP and SP datasets, except for the NWRS8 instance, which was the only instance created by a pseudorandom instance generator.
- **Shaw** [7]. Dataset with 25 20×20 instances, without information about the method used to generate it.
- **SP** [7]. Set of four instances, 25×25, 50×50, 75×75 and 100×100, generated by the methods used to generate the GP dataset.
- **SCOOP** [42]. Set of 24 real instances of two sawmilling companies.
- ***Faggioli & Bentivoglio*** [12]. Set of 300 instances created by a pseudorandom instance generator with *m* = 10, 15, 20, 25, 30, 40 and *n* = 10, 20, 30, 40, 50. Each *m* × *n* combination has 10 instances.
- ***Chu & Stuckey*** [17]. Set of 200 instances more difficult than those proposed in [7], with 30, 40, 50, 75, 100 or 125 patterns and pieces. For each combination of numbers of patterns and pieces, sets of five instances with 2, 4, 6, 8 and 10 pieces per pattern were created by a pseudorandom instance generator, totaling 25 instances by dimension.
- ***Carvalho & Soma*** [9]. Set of instances with larger dimensions, 150×150, 175×175 and 200×200, created by the instance generator mechanism developed by *Chu & Stuckey* [17]. Ten instances of 2, 4, 6, 8 and 10 pieces per pattern were created for each dimension, totaling 150 instances.

To assess the performance of the methods for datasets larger than those currently available in the literature, we created instances with dimensions of 400×400, 600×600, 800×800 and 1000×1000 using the instance generator developed by *Chu & Stuckey* [17]. In contrast to the instances proposed by *Chu & Stuckey* and by *Carvalho & Soma*, we analyzed a larger number of pieces by pattern than 2, 4, 6, 8 and 10 to assess the performance of the heuristics for graphs of different densities to identify possible convergence between methods in relation to the value of the solution for a specific density. To obtain variation in graph density, ten instances of 2, 4, 6, 8, 10, 14, 18, 20, 24, 28, 30 and 34 pieces by pattern were created in the 400×400 matrices. In the other datasets, the instances followed the same proportions but included 38 and 40 pieces by pattern in the 600×600 instances, 38, 40, 44, 48 and 50 in the 800×800 instances and 38, 40, 44, 48, 50 and 54 in the 1000×1000 instances. The instance of dimension 1000 analyzed here is 5 times larger than datasets used in state-of-art methods experiments, representing a 25-fold increase in input size. We generated 610 new instances, totaling 1330 experiments. The datasets are available in Supporting Information S1 Dataset—MOSP Instances Files.

The codes of the *MCNh* and *HBF_2r_* methods were the same as those developed by *Carvalho & Soma* [9], and both were written in ANSI-C language compiled with gcc 5.4.0 and the -O3 optimization option. The preprocessing operation by pattern dominance was also run in these methods [4]. The original code of the *Chu & Stuckey* method, which was written in ANSI-C language, was compiled and run as recommended by the authors to obtain the optimal values. All experiments were performed in the same computing environment. In cases of multiple instances, the mean of the results was analyzed.

### Results for the *First Constraint Modelling Challenge* dataset

Table 2 outlines the results for the *First Constraint Modelling Challenge* dataset, whose density ranges from 0.196 to 0.995. The Shaw subset has 25 instances, and all others have only one. The column D is the MOSP graph density, OPT is the value of the optimal solution in *MNOS*, the Time values are expressed in milliseconds, Sol. is the value of the method solution in *MNOS*. The Total row contains the sum of values of the respective column, except for the column %gap, whose value is the percent gap of all solutions obtained by the method in relation to all optimal solutions. Optimal results are bolded.

**Table 2:** Results for the *First Constraint Modelling Challenge* dataset.

Dataset	D	OPT	*MCNh*			*HBF_2r_*			*PieceRank*		
			Time	Sol.	%gap	Time	Sol.	%gap	Time	Sol.	%gap
GP1 50×50	0.980	45	0.00	**45**	0.00	0.04	**45**	0.00	0.01	**45**	0.00
GP2 50×50	0.940	40	0.00	**40**	0.00	0.10	**40**	0.00	0.01	**40**	0.00
GP3 50×50	0.954	40	0.00	**40**	0.00	0.07	**40**	0.00	0.01	41	2.50
GP4 50×50	0.820	30	0.00	**30**	0.00	0.03	**30**	0.00	0.00	31	3.33
GP5 100×100	0.995	95	0.01	96	1.05	0.30	96	1.05	0.17	96	1.05
GP6 100×100	0.934	75	0.01	**75**	0.00	0.34	**75**	0.00	0.11	**75**	0.00
GP7 100×100	0.933	75	0.01	**75**	0.00	0.44	**75**	0.00	0.11	**75**	0.00
GP8 100×100	0.831	60	0.01	**60**	0.00	0.40	61	1.67	0.08	61	1.67
Miller 40×20	0.526	13	0.00	**13**	0.00	0.02	**13**	0.00	0.00	**13**	0.00
NWRS1 20×10	0.378	3	0.00	**3**	0.00	0.00	**3**	0.00	0.00	**3**	0.00
NWRS2 20×10	0.489	4	0.00	**4**	0.00	0.00	**4**	0.00	0.00	**4**	0.00
NWRS3 25×15	0.505	7	0.00	**7**	0.00	0.00	**7**	0.00	0.00	**7**	0.00
NWRS4 25×15	0.610	7	0.00	**7**	0.00	0.00	**7**	0.00	0.00	**7**	0.00
NWRS5 30×20	0.742	12	0.00	**12**	0.00	0.01	**12**	0.00	0.00	**12**	0.00
NWRS6 30×20	0.753	12	0.00	**12**	0.00	0.01	**12**	0.00	0.00	**12**	0.00
NWRS7 60×25	0.453	10	0.00	**10**	0.00	0.01	**10**	0.00	0.00	**10**	0.00
NWRS8 60×25	0.697	16	0.00	**16**	0.00	0.03	**16**	0.00	0.00	**16**	0.00
Shaw 20×20	0.665	13.68	0.00	14.00	2.34	0.00	13.76	0.58	0.00	13.92	1.75
SP1 25×25	0.260	9	0.00	**9**	0.00	0.00	**9**	0.00	0.00	**9**	0.00
SP2 50×50	0.210	19	0.00	23	21.05	0.04	**19**	0.00	0.00	22	15.79
SP3 75×75	0.196	34	0.00	37	8.82	0.21	35	2.94	0.00	40	17.65
SP4 100×100	0.212	53	0.00	57	7.55	0.64	**53**	0.00	0.00	57	7.55
	**Total**	672.68	0.04	685.00	1.83	2.68	675.76	0.46	0.52	689.92	2.56

Regarding the solution quality, *HBF_2r_* obtained optimal solutions in 81.8% of instances, *MCNh* in 77.27% and *PieceRank* in 63.63%. In the GP dataset, all non-optimal results obtained by *PieceRank* opened only 1 stack more than the optimal solution, and the worst solutions were observed in the SP dataset, with 15% and 17% gaps. Overall, although *HBF_2r_* was the slowest method, it obtained the lowest gap (2.68 s, 0.46%), *MCNh* was the fastest (0.04 ms, 1.83%), and *PieceRank* had the worst gap (0.52 ms and 2.56 %gap). *MCNh* maintained a more consistent time for a given dimension, in contrast to *HBF_2r_* and *PieceRank*, which showed greater variations in denser instances, such as the instances GP5 to GP8.

### Results for the SCOOP dataset

**Fig 6:**
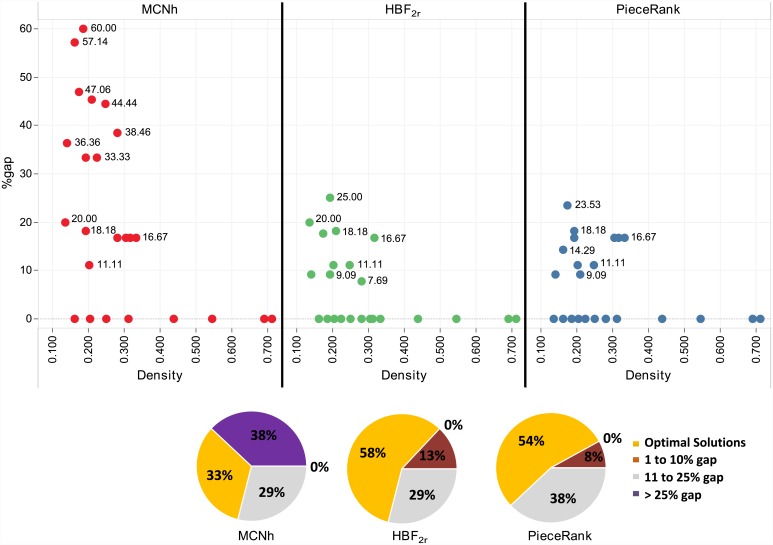
Results for the SCOOP dataset.

The graph shown in Fig 6 contains the results of 24 individual instances of the SCOOP dataset. The densities of the graphs range from 0.163 to 0.711. The graph is divided into three columns with the results of each method. The x-axis contains the densities of the graphs, and the y-axis contains the gap between the instance and the optimal solution. The pie charts illustrate, for each method, the percentage of solutions for a given gap.

*HBF_2r_* obtained optimal solutions in 58% of cases, *PieceRank* in 54% and *MCNh* in 33%. *HBF_2r_* and *PieceRank* found solutions with only one more stack than the optimal solution in 29% of cases and *MCNh* in 25% of cases. *MCNh* found gaps larger than 25% in 38% of instances. The results showed that the gaps of the *MCNh* solutions presents values larger and more dispersed than those of the other methods. *MCNh* had the largest gap (60%), whereas the largest gaps of *HBF_2r_* and *PieceRank* were 25% and 23.05%, respectively. Considering the total value of the optimal solutions (186), *HBF_2r_* and *PieceRank* obtained gaps of 8.06% (0.23 ms) and 8.60% (0.04 ms), respectively, and *MCNh* obtained a gap of 25.27% (0.02 ms).

### Results for the *Faggioli & Bentivoglio* dataset

**Fig 7:**
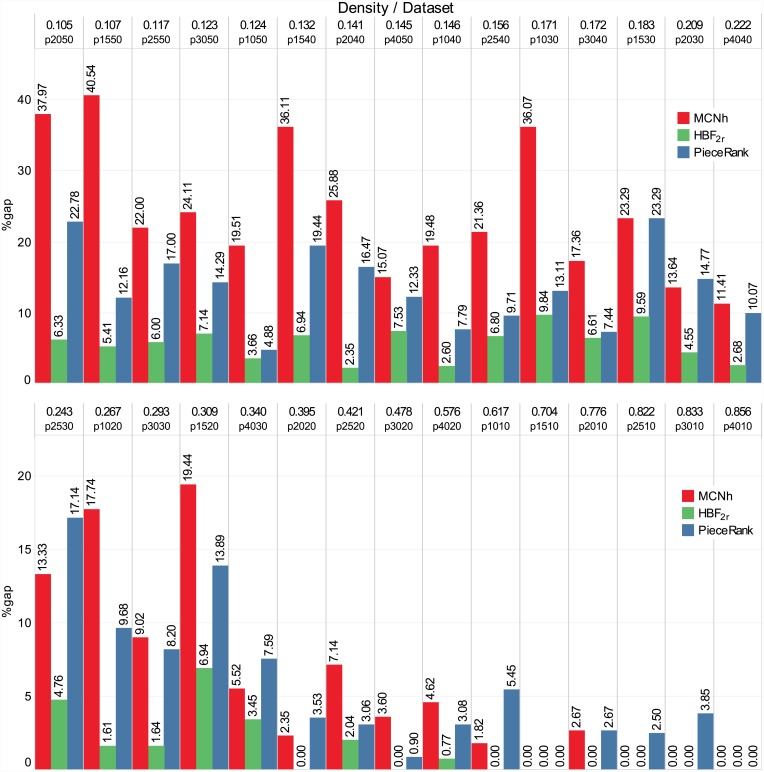
Results for the *Faggioli & Bentivoglio* dataset.

The results for the *Faggioli & Bentivoglio* dataset are shown in Fig 7. The instances of this dataset are divided into groups according to their dimension, and each group has 10 instances. The instances are organized in non-decreasing order of MOSP graph density. The text at the top of the bar indicates the gap between the method solution and the optimal solution values. The name of each group describes their dimension (e.g., p1020n has 10 patterns and 20 pieces). The density of the graphs ranges from 0.105 to 0.856. The runtime of the methods is very low: 0.16 ms for *HBF_2r_* and 0.00 ms for *MCNh* and *PieceRank*. For all methods, gaps smaller than 10% are observed in instances with density ranging from 0.341 to 0.822. The largest gaps occur in less dense instances (density ranging from 0.105 to 0.300). In total, *HBF_2r_* had a 3.69% gap, *PieceRank* 9.46% and *MCNh* 14.37%. *PieceRank* obtained better solutions than *MCNh* at the same computing cost for 63.33% of the instances.

### Results for the *Chu & Stuckey* and *Carvalho & Soma* datasets

**Fig 8:**
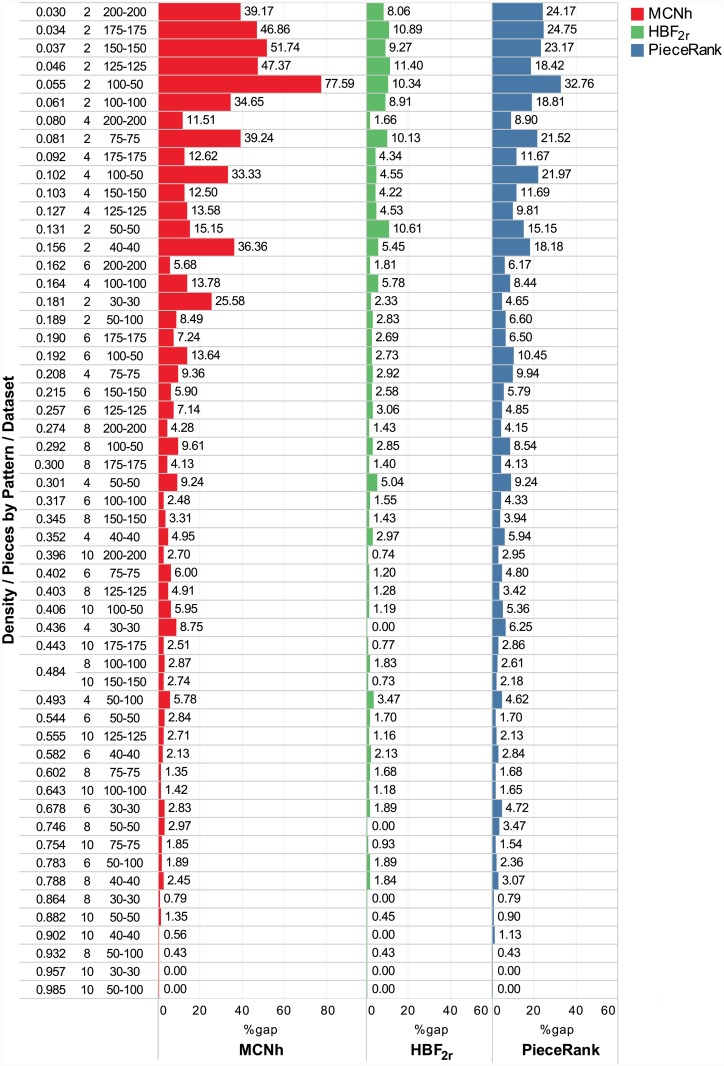
Results for the *Chu & Stuckey* (30×30 to 125×125) and *Carvalho & Soma* (150×150 to 200×200) datasets.

Fig 8 contains the results for the *Chu & Stuckey* and *Carvalho & Soma* datasets. These datasets are considered more difficult because their graphs lack specific structures that facilitate their solution [4]. The density of these datasets ranges from 0.030 to 0.985. Instances are organized into groups according to their size and maximum number of pieces by pattern. Each dimension of the *Chu & Stuckey* dataset has 5 instances per group, and each dimension of the *Carvalho & Soma* has 10. The text at the top of the bar indicates the gap between the method solution and the optimal solution values.

*HBF_2r_* obtained the best results in 85% of the *Chu & Stuckey* instances and in all *Carvalho & Soma* instances. Similar to the previously analyzed datasets of instances, the worst gaps occurred in low density instances (0.30 to 0.181). This performance was observed by *Carvalho & Soma* [9], who suggest as an explanation the fact that, in very sparse graphs, new stacks can be prematurely opened for each sequenced pattern. In *Chu & Stuckey* instances, *PieceRank* found better solutions than *MCNh* in 57.50% of cases, worse solutions in 27.50% of cases and equal solutions in 15.00% of cases. Conversely, in *Carvalho & Soma* instances, *PieceRank* found better solutions than *MCNh* in 66.67%, worse solutions in 26.267% of cases and equal solutions in 6.66% of cases. Considering the total sum of optimal solutions, *HBF_2r_* obtained a 2.25% gap (219.69 ms), *PieceRank* 5.74% (0.33 ms) and *MCNh* 7.58% (0.25 ms).

### Results for the new datasets

**Fig 9:**
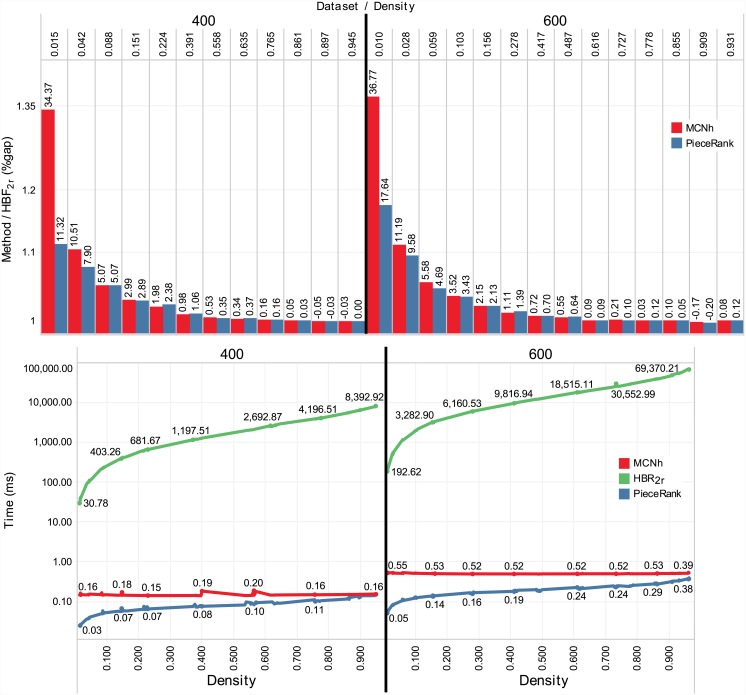
Results for the new 400×400 and 600×600 datasets. For improved visualization, in all graphs, the y-axis is in the logarithmic scale.

Fig 9 contains the results of the proposed datasets with instances with dimensions of 400×400 and 600×600. Due to the long computing time required by the exact method, the *HBF_2r_* results are used as a reference to calculate the gap. The *HBF_2r_* solutions are not proven optimal. The bar graph shows the mean value of the solutions obtained by the method divided by the mean value of the solutions obtained by *HBF_2r_*. The text at the top of the bar indicates the gap between the method solution and the value of the *HBF_2r_* solution.

*PieceRank* had obtained gaps equal to or smaller than *MCNh* in 58.33% of cases in the set of 400×400 instances and in 71.43% of cases in the set of 600×600 instances. The gap between solutions became smaller than 1% (e.g., 400-18 onward) with increasing density. A possible explanation for this performance is the tendency toward a complete graph (density 1.00) with the increase in graph density. This translates into patterns with strong connectivity with other patterns because they have many pieces in common. This condition somewhat reduces the variability of the solutions, whose values are near the upper limit.

Considering the total gap, *PieceRank* obtained a value of 1.49% and *MCNh* 1.94%. The *MCNh* runtime showed little variation, even in denser sets, in contrast to *PieceRank*, whose runtime gradually increased with graph density. Despite this increase, *PieceRank* obtained the shortest runtimes among all methods. In some cases, *MCNh* obtained a time approximately 9 times longer than that of *PieceRank* (e.g., 600-600-2, density 0.010). Conversely, the *HBF_2r_* runtime increased rapidly with the size of instances, requiring approximately 80 hours to solve all sets, well above the runtimes of *PieceRank* (3.96 s) and *MCNh* (9.51 s).

Table 3 outlines the results for the proposed dataset with 800×800 and 1000×1000 instances. Due to the runtime required, the *HBF_2r_* method was disregarded in the analyses of these datasets, and the *MCNh* results were used as the reference for the gap of *PieceRank* solutions. The PbP column contains the number of Pieces by Pattern, D is the MOSP graph density, the Time values are expressed in milliseconds, Sol. is the value of the method solution in *MNOS*. The Total row contains the sum of the values in the respective column, except for the column %gap, whose value is the total gap between all *PieceRank* solutions and all *MCNh* solutions. The best values are bolded.

**Table 3:** Results for the new datasets of 800×800 and 1000×1000 instances.

PbP	D	800×800					PbP	D	1000×1000				
		*MCNh*		*PieceRank*					*MCNh*		*PieceRank*		
		Time	Sol.	Time	Sol.	%gap			Time	Sol.	Time	Sol.	%gap
2	0.009	1.49	190.40	0.09	**161.50**	-15.18	2	0.007	3.37	237.30	0.13	**200.80**	-15.38
4	0.021	1.47	364.00	0.15	**354.70**	-2.55	4	0.017	3.31	449.40	0.22	**439.30**	-2.25
6	0.044	1.45	506.30	0.20	**504.20**	-0.41	6	0.036	3.40	635.80	0.29	**630.90**	-0.77
8	0.077	1.42	599.60	0.22	**596.60**	-0.50	8	0.063	3.23	**748.20**	0.34	748.60	0.05
10	0.117	1.38	**658.40**	0.25	658.50	0.02	10	0.095	3.18	822.30	0.37	**821.20**	-0.13
14	0.216	1.36	**720.30**	0.28	720.50	0.03	14	0.178	3.12	899.20	0.42	**899.10**	-0.01
18	0.336	1.34	**748.70**	0.32	749.80	0.15	18	0.277	3.11	936.00	0.48	**935.10**	-0.10
20	0.394	1.34	**757.80**	0.34	758.40	0.08	20	0.329	3.13	**945.80**	0.50	945.90	0.01
24	0.514	1.35	**770.50**	0.37	770.60	0.01	24	0.438	3.14	**962.50**	0.55	962.90	0.04
28	0.626	1.35	778.40	0.42	**778.00**	-0.05	28	0.544	3.60	972.40	0.62	**972.10**	-0.03
30	0.678	1.36	**781.10**	0.44	781.20	0.01	30	0.594	3.50	976.10	0.65	**975.30**	-0.08
34	0.767	1.52	**785.30**	0.49	**785.30**	0.00	34	0.685	3.30	981.80	0.71	**981.60**	-0.02
38	0.836	1.37	**788.80**	0.53	788.90	0.01	38	0.765	3.28	985.10	0.77	**985.00**	-0.01
40	0.864	1.37	789.90	0.57	**789.80**	-0.01	40	0.797	3.29	986.90	0.80	**986.70**	-0.02
44	0.911	1.41	792.10	0.62	**791.90**	-0.03	44	0.855	3.22	989.30	0.88	**989.20**	-0.01
48	0.944	1.39	**793.20**	0.69	**793.20**	0.00	48	0.901	3.26	**991.40**	0.98	**991.40**	0.00
50	0.957	1.37	794.10	0.74	**793.80**	-0.04	50	0.918	3.21	991.90	1.04	**991.60**	-0.03
	**Total**	23.74	11618.89	6.71	11576.90	-0.36	54	0.946	3.20	993.30	1.14	**992.90**	-0.04
								**Total**	58.89	15504.70	10.88	15449.60	-0.36

Regarding solution quality, *PieceRank* obtained results equal to or better than *MCNh* for 52.94% of the sets of 800×800 dimension and 83.33% of the sets of 1000×1000 dimension. The best solutions were observed in sets with instances of up to 2 pieces by pattern, with a -15.18% gap in instances of 800×800 dimension and a -15.38% gap in instances of 1000×1000 dimension. The gaps were small, even for solutions in which *PieceRank* had worse results than *MCNh*, and the worst cases were 0.15% in 800-18 instances and 0.05% in 1000-8 instances.

**Fig 10:**
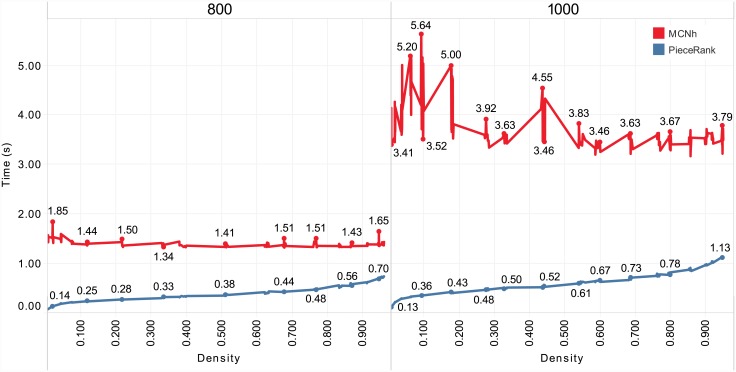
Runtime results for the new 800×800 and 1000×1000 datasets.

The graph in Fig 10 shows in detail the runtimes of each method at each density. *PieceRank* had considerably shorter runtimes; for example, in the 1000-1000-2 dataset, *MCNh* required 3.37 s (237.30 solution) and *PieceRank* 0.13 ms (200.80 solution), that is, a difference in runtime of approximately 26 times.

Similarly, for the datasets of 400×400 and 600×600 instances, *MCNh* was more affected by dataset dimension than by graph density because its runtimes remained highly constant despite the increase in graph density. Conversely, *PieceRank* was more sensitive to density, as shown by the increase in runtime. Overall, *MCNh* (82.63 s) required a runtime nearly 5 times longer than that of *PieceRank* (17.59 s) for these datasets.

## Conclusion and future studies

Computing large instances is required in specific modeling problems similar to the MOSP, but most current methods were not designed for this task and have proved unviable. Based on measures and methods used in network science, we proposed the *PageRank*-based heuristic *PieceRank* to solve large MOSP instances in this study. To validate *PieceRank*, computational experiments were performed to analyze datasets commonly used in the literature, in addition to new, larger datasets. The proposed heuristic was compared with state-of-the-art methods in terms of solution quality and runtime.

The findings highlighted the ability of *PieceRank* to obtain quality solutions in short runtimes. The heuristic performed best with larger and less dense instances, but quality solutions were also obtained in smaller instances, albeit in fewer cases. We observed that for *First Constraint Modelling Challenge* dataset, although *PieceRank* obtained optimal solutions in several cases, it had the worst results in terms of quality. *PieceRank* showed better performances than *MCNh* for the *Chu & Stuckey* and *Carvalho & Soma* instances.

With real SCOOP datasets, whose instances were mostly smaller than 50, *PieceRank* performed similarly to *HBF_2r_* and with better quality than *MCNh*. The analysis as a function of graph density showed that the largest gaps occurred for graphs with densities lower than 0.250 for all methods. For graphs with these characteristics, the quality of *PieceRank* solutions was less compromised than that of *MCNh* solutions. In the new datasets, which have instances of larger dimensions, *PieceRank* was competitive and a better alternative than *MCNh* for the construction of initial solutions, especially for less dense graphs. *PieceRank* had the shortest runtime in all cases and better results than *MCNh* in most cases. The analysis as a function of MOSP graph density showed that, for specific densities, the gap between the methods was less than 1% (e.g., 0.558 in the 400×400 dataset and 0.417 in the 600×600 dataset). Our heuristic effectively solved large instances with the shortest runtime of all methods.

In this study, we innovated by using centrality as a measure instead of directly using node degree to conduct the search. In addition to the advantage of fast calculation, centrality provides a more accurate view of graph node importance than degree-based criteria. This feature enabled us to identify the most promising graph regions to start the sequencing and may improve solution quality. We also expanded the range of *PageRank* applications. *PageRank* was found to be viable for solving similar graph-modeled manufacturing problems.

The graph density analyses indicated cases of convergence, which were characterized by a very small difference in the value of solutions between methods. This small difference may justify the use of the fastest method. We observed that, in larger instances, convergence tended to occur from lower densities. This finding may indicate that the triviality of the MOSP solution is also associated with other structures not yet identified, in addition to the structures already known and cited herein. This analysis paves the way for future studies because we suspect that the most suitable method to solve a given MOSP graph may be determined by the graph features and use of classifiers (e.g., decision tree, neural network).

In preliminary tests conducted to define the *PieceRank* parameters, we noted a *damping factor* effect on solution quality and on runtime, particularly in large instances. We observed that, at densities lower than 0.600, a *damping factor* ranging from 0.35 to 0.55 helped obtain the best solutions, whereas a *damping factor* ranging from 0.60 to 0.95 yielded shorter runtimes. At higher densities, the *damping factor* effect was also observed but tended to be less significant. Studies involving the application of *PieceRank* auto-parameterization methods may help define the most suitable *damping factor* for the best trade-off between solution quality and runtime. Other study possibilities involve applying *PieceRank* in weighted MOSP graphs, as well to construct initial solutions for subsequent improvement by enumerative methods or by local search. The use of centrality and other available measures in network science to solve the MOSP and other types of graph-modeled combinatorial problems, are also promising themes for study.

## Supporting information

S1 Table Detailed Results of Experiments. Contains the tables with the results of the experiments of all analyzed datasets to *Chu & Stuckey* (OPT), *HBF*_2*r*_, *MCNh*, *PieceRank*, *Yuen* and *Ashikaga & Soma* methods.
(PDF)

S1 Dataset MOSP Instance Files. Contains the files and links to get the datasets used in the experiments.
(ZIP)
