# Supplementary material for: A PageRank-based heuristic for the minimization of open stacks problem
Source: PLoS One. 2018 Aug 30;13(8):e0203076. doi: 10.1371/journal.pone.0203076 (PMC6117050; doi:10.1371/journal.pone.0203076)
Supplement: S1 Table — Contains the tables with the results of the experiments of all analyzed datasets to Chu & Stuckey (OPT), HBF2r, MCNh, PieceRank, Yuen and Ashikaga & Soma methods. (PDF) [file pone.0203076.s001.pdf]

S1 Table - Detailed Results of Experiments

Frinhani, Carvalho & Soma - A PageRank-based heuristic for the minimization of open stacks problem (July, 2018)

- This Supporting Information contains the tables with the detailed results of the experiments.
- The references of the methods and datasets used are found at the end of this document.
- In Ashikaga & Soma, solutions highlighted with "*" have an error. The value is less than the optimal proven solution.
- Time (milliseconds), Solution (maximum number of open stacks)

KEY

D: Density of MOSP graph

OPT: Optimal Solution **(**Chu & Stuckey, 2009)

***** : Solutions with error

First Constraint Modelling Challenge

Dataset	D	OPT	HBF2r			MCNh			PieceRank			Yuen3			Ashikaga & Soma		
			Time	Solution	%Gap	Time	Solution	%Gap	Time	Solution	%Gap	Time	Solution	%Gap	Time	Solution	%Gap
GP1	0.980	45	0.04	45	0.00	0.00	45	0.00	0.01	45	0.00	0.00	48	6.67	0.00	50	11.11
GP2	0.940	40	0.10	40	0.00	0.00	40	0.00	0.01	40	0.00	0.00	48	20.00	0.00	50	25.00
GP3	0.954	40	0.07	40	0.00	0.00	40	0.00	0.01	41	2.50	0.00	45	12.50	0.00	50	25.00
GP4	0.820	30	0.03	30	0.00	0.00	30	0.00	0.00	31	3.33	0.00	44	46.67	0.00	50	66.67
GP5	0.995	95	0.30	96	1.05	0.01	96	1.05	0.17	96	1.05	0.00	100	5.26	0.00	100	5.26
GP6	0.934	75	0.34	75	0.00	0.01	75	0.00	0.11	75	0.00	0.00	99	32.00	0.00	100	33.33
GP7	0.933	75	0.44	75	0.00	0.01	75	0.00	0.11	75	0.00	0.00	89	18.67	0.00	100	33.33
GP8	0.831	60	0.40	61	1.67	0.01	60	0.00	0.08	61	1.67	0.00	78	30.00	0.00	100	66.67
Miller	0.526	13	0.02	13	0.00	0.00	13	0.00	0.00	13	0.00	0.00	13	0.00	0.00	7 *	-46.15
NWRS1	0.378	3	0.00	3	0.00	0.00	3	0.00	0.00	3	0.00	0.00	3	0.00	0.00	5	66.67
NWRS2	0.489	4	0.00	4	0.00	0.00	4	0.00	0.00	4	0.00	0.00	6	50.00	0.00	7	75.00
NWRS3	0.505	7	0.00	7	0.00	0.00	7	0.00	0.00	7	0.00	0.00	8	14.29	0.00	10	42.86
NWRS4	0.610	7	0.00	7	0.00	0.00	7	0.00	0.00	7	0.00	0.00	8	14.29	0.00	12	71.43
NWRS5	0.742	12	0.01	12	0.00	0.00	12	0.00	0.00	12	0.00	0.00	13	8.33	0.00	14	16.67
NWRS6	0.753	12	0.01	12	0.00	0.00	12	0.00	0.00	12	0.00	0.00	14	16.67	0.00	15	25.00
NWRS7	0.453	10	0.01	10	0.00	0.00	10	0.00	0.00	10	0.00	0.00	15	50.00	0.00	16	60.00
NWRS8	0.697	16	0.03	16	0.00	0.00	16	0.00	0.00	16	0.00	0.00	18	12.50	0.00	18	12.50
Shaw	0.665	13.68	0.00	13.76	0.58	0.00	14.00	2.34	0.00	13.92	1.75	0.00	15.08	10.23	0.00	19.00	38.89
SP1	0.260	9	0.00	9	0.00	0.00	9	0.00	0.00	9	0.00	0.00	10	11.11	0.00	11	22.22
SP2	0.210	19	0.04	19	0.00	0.00	23	21.05	0.00	22	15.79	0.00	27	42.11	0.00	30	57.89
SP3	0.196	34	0.21	35	2.94	0.00	37	8.82	0.00	40	17.65	0.00	42	23.53	0.00	56	64.71
SP4	0.212	53	0.64	53	0.00	0.00	57	7.55	0.00	57	7.55	0.00	69	30.19	0.00	79	49.06
	**Total**	672.68	2.68	675.76	0.46	0.04	685.00	1.83	0.52	689.92	2.56	0.00	812.08	20.72	0.00	892.00	32.60

SCOOP Project																	
**Dataset**	**Densidade**	**OPT**	**HBF2r**			**MCNh**			**PieceRank**			**Yuen3**			**Ashikaga & Soma**		
			**Time**	**Solution**	**%Gap**	**Time**	**Solution**	**%Gap**	**Time**	**Solution**	**%Gap**	**Time**	**Solution**	**%Gap**	**Time**	**Solution**	**%Gap**
SCOOP-A_AP-9.d_10	0.333	6	0.00	6	0.00	0.00	7	16.67	0.00	7	16.67	0.00	7	16.67	0.00	8	33.33
SCOOP-A_AP-9.d_11	0.224	6	0.00	6	0.00	0.00	8	33.33	0.00	6	0.00	0.00	8	33.33	0.00	13	116.67
SCOOP-A_AP-9.d_3	0.250	6	0.00	6	0.00	0.00	6	0.00	0.00	6	0.00	0.00	6	0.00	0.00	9	50.00
SCOOP-A_AP-9.d_6	0.137	5	0.00	6	20.00	0.00	6	20.00	0.00	5	0.00	0.00	6	20.00	0.00	10	100.00
SCOOP-A_FA_1	0.192	12	0.05	15	25.00	0.00	16	33.33	0.00	14	16.67	0.00	15	25.00	0.00	52	333.33
SCOOP-A_FA_11	0.140	11	0.03	12	9.09	0.00	15	36.36	0.00	12	9.09	0.00	16	45.45	0.00	47	327.27
SCOOP-A_FA_12	0.247	9	0.01	10	11.11	0.00	13	44.44	0.00	10	11.11	0.00	14	55.56	0.00	32	255.56
SCOOP-A_FA_13	0.174	17	0.07	20	17.65	0.01	25	47.06	0.00	21	23.53	0.00	26	52.94	0.00	81	376.47
SCOOP-A_FA_15	0.203	9	0.01	10	11.11	0.00	10	11.11	0.00	10	11.11	0.00	13	44.44	0.00	33	266.67
SCOOP-A_FA_2	0.193	11	0.01	12	9.09	0.00	13	18.18	0.00	13	18.18	0.00	14	27.27	0.00	32	190.91
SCOOP-A_FA_6	0.281	13	0.01	14	7.69	0.00	18	38.46	0.00	13	0.00	0.00	16	23.08	0.00	35	169.23
SCOOP-A_FA_8	0.209	11	0.02	13	18.18	0.00	16	45.45	0.00	12	9.09	0.00	15	36.36	0.00	31	181.82
SCOOP-B_12F18_11	0.305	6	0.00	6	0.00	0.00	7	16.67	0.00	7	16.67	0.00	6	0.00	0.00	11	83.33
SCOOP-B_12M18_12	0.316	6	0.00	7	16.67	0.00	7	16.67	0.00	7	16.67	0.00	6	0.00	0.00	18	200.00
SCOOP-B_18AB1_32	0.545	6	0.00	6	0.00	0.00	6	0.00	0.00	6	0.00	0.00	6	0.00	0.00	9	50.00
SCOOP-B_18CR1_33	0.163	4	0.00	4	0.00	0.00	4	0.00	0.00	4	0.00	0.00	4	0.00	0.00	9	125.00
SCOOP-B_22X18_50	0.689	10	0.00	10	0.00	0.00	10	0.00	0.00	10	0.00	0.00	10	0.00	0.00	10	0.00
SCOOP-B_23B25_52	0.186	5	0.00	5	0.00	0.00	8	60.00	0.00	5	0.00	0.00	6	20.00	0.00	12	140.00
SCOOP-B_39Q18_82	0.711	5	0.00	5	0.00	0.00	5	0.00	0.00	5	0.00	0.00	6	20.00	0.00	9	80.00
SCOOP-B_42F22_93	0.311	5	0.00	5	0.00	0.00	5	0.00	0.00	5	0.00	0.00	6	20.00	0.00	5	0.00
SCOOP-B_CARLET_137	0.439	5	0.00	5	0.00	0.00	5	0.00	0.00	5	0.00	0.00	6	20.00	0.00	8	60.00
SCOOP-B_CUC28A_138	0.280	6	0.00	6	0.00	0.00	7	16.67	0.00	6	0.00	0.00	8	33.33	0.00	22	266.67
SCOOP-B_GTM18A_139	0.205	5	0.00	5	0.00	0.00	5	0.00	0.00	5	0.00	0.00	5	0.00	0.00	13	160.00
SCOOP-B_REVAL_145	0.163	7	0.02	7	0.00	0.00	11	57.14	0.00	8	14.29	0.00	9	28.57	0.00	36	414.29
	**Total**	186.00	0.23	201	8.06	0.02	233	25.27	0.04	202	8.60	0.00	234	25.81	0.00	545	171.14

Faggioli & Bentivoglio																		
Dataset	Densidade	OPT	HBF2r			MCNh			PieceRank			Yuen3			Ashikaga & Soma		
			Time	Solution	%Gap	Time	Solution	%Gap	Time	Solution	%Gap	Time	Solution	%Gap	Time	Solution	%Gap
p1010n	0.617	5.50	0.00	5.50	0.00	0.00	5.60	1.82	0.00	5.80	5.45	0.00	6.00	9.09	0.00	7.50	36.36
p1020n	0.267	6.20	0.00	6.30	1.61	0.00	7.30	17.74	0.00	6.80	9.68	0.00	7.20	16.13	0.00	7.40	19.35
p1030n	0.171	6.10	0.00	6.70	9.84	0.00	8.30	36.07	0.00	6.90	13.11	0.00	7.00	14.75	0.00	14.20	132.79
p1040n	0.146	7.70	0.00	7.90	2.60	0.00	9.20	19.48	0.00	8.30	7.79	0.00	8.20	6.49	0.00	17.20	123.38
p1050n	0.124	8.20	0.00	8.50	3.66	0.00	9.80	19.51	0.00	8.60	4.88	0.00	8.60	4.88	0.00	19.00	131.71
p1510n	0.704	6.60	0.00	6.60	0.00	0.00	6.60	0.00	0.00	6.60	0.00	0.00	7.00	6.06	0.00	8.80	33.33
p1520n	0.309	7.20	0.00	7.70	6.94	0.00	8.60	19.44	0.00	8.20	13.89	0.00	9.00	25.00	0.00	12.10	68.06
p1530n	0.183	7.30	0.00	8.00	9.59	0.00	9.00	23.29	0.00	9.00	23.29	0.00	9.20	26.03	0.00	14.80	102.74
p1540n	0.132	7.20	0.00	7.70	6.94	0.00	9.80	36.11	0.00	8.60	19.44	0.00	9.40	30.56	0.00	17.60	144.44
p1550n	0.107	7.40	0.00	7.80	5.41	0.00	10.40	40.54	0.00	8.30	12.16	0.00	9.00	21.62	0.00	19.40	162.16
p2010n	0.776	7.50	0.00	7.50	0.00	0.00	7.70	2.67	0.00	7.70	2.67	0.00	7.90	5.33	0.00	8.00	6.67
p2020n	0.395	8.50	0.00	8.50	0.00	0.00	8.70	2.35	0.00	8.80	3.53	0.00	10.50	23.53	0.00	14.40	69.41
p2030n	0.209	8.80	0.00	9.20	4.55	0.00	10.00	13.64	0.00	10.10	14.77	0.00	11.20	27.27	0.00	14.10	60.23
p2040n	0.141	8.50	0.00	8.70	2.35	0.00	10.70	25.88	0.00	9.90	16.47	0.00	10.70	25.88	0.00	14.40	69.41
p2050n	0.105	7.90	0.01	8.40	6.33	0.00	10.90	37.97	0.00	9.70	22.78	0.00	10.40	31.65	0.00	14.70	86.08
p2510n	0.822	8.00	0.00	8.00	0.00	0.00	8.00	0.00	0.00	8.20	2.50	0.00	8.50	6.25	0.00	8.20	2.50
p2520n	0.421	9.80	0.00	10.00	2.04	0.00	10.50	7.14	0.00	10.10	3.06	0.00	12.30	25.51	0.00	15.50	58.16
p2530n	0.243	10.50	0.01	11.00	4.76	0.00	11.90	13.33	0.00	12.30	17.14	0.00	13.80	31.43	0.00	20.20	92.38
p2540n	0.156	10.30	0.01	11.00	6.80	0.00	12.50	21.36	0.00	11.30	9.71	0.00	13.70	33.01	0.00	18.80	82.52
p2550n	0.117	10.00	0.01	10.60	6.00	0.00	12.20	22.00	0.00	11.70	17.00	0.00	13.60	36.00	0.00	15.90	59.00
p3010n	0.833	7.80	0.00	7.80	0.00	0.00	7.80	0.00	0.00	8.10	3.85	0.00	8.50	8.97	0.00	8.80	12.82
p3020n	0.478	11.10	0.01	11.10	0.00	0.00	11.50	3.60	0.00	11.20	0.90	0.00	13.70	23.42	0.00	15.80	42.34
p3030n	0.293	12.20	0.01	12.40	1.64	0.00	13.30	9.02	0.00	13.20	8.20	0.00	14.80	21.31	0.00	21.70	77.87
p3040n	0.172	12.10	0.01	12.90	6.61	0.00	14.20	17.36	0.00	13.00	7.44	0.00	15.90	31.40	0.00	23.60	95.04
p3050n	0.123	11.20	0.01	12.00	7.14	0.00	13.90	24.11	0.00	12.80	14.29	0.00	15.60	39.29	0.00	19.50	74.11
p4010n	0.856	8.40	0.00	8.40	0.00	0.00	8.40	0.00	0.00	8.40	0.00	0.00	8.90	5.95	0.00	8.40	0.00
p4020n	0.576	13.00	0.01	13.10	0.77	0.00	13.60	4.62	0.00	13.40	3.08	0.00	15.30	17.69	0.00	15.90	22.31
p4030n	0.340	14.50	0.02	15.00	3.45	0.00	15.30	5.52	0.00	15.60	7.59	0.00	18.40	26.90	0.00	23.00	58.62
p4040n	0.222	14.90	0.02	15.30	2.68	0.00	16.60	11.41	0.00	16.40	10.07	0.00	18.80	26.17	0.00	27.20	82.55
p4050n	0.145	14.60	0.03	15.70	7.53	0.00	16.80	15.07	0.00	16.40	12.33	0.00	19.70	34.93	0.00	29.10	99.32
	**Total**	279.00	0.16	289.30	3.69	0.00	319.10	14.37	0.00	305.40	9.46	0.00	342.80	22.87	0.00	475.20	70.32

Chu & Stuckey

Dataset	Densidade	OPT	HBF2r			MCNh			PieceRank			Yuen3			Ashikaga & Soma		
			Time	Solution	%Gap	Time	Solution	%Gap	Time	Solution	%Gap	Time	Solution	%Gap	Time	Solution	%Gap
Random-30-30-2	0.181	8.60	0.01	8.80	2.33	0.00	10.80	25.58	0.00	9.00	4.65	0.00	12.80	48.84	0.00	19.00	120.93
Random-30-30-4	0.436	16.00	0.01	16.00	0.00	0.00	17.40	8.75	0.00	17.00	6.25	0.00	19.80	23.75	0.00	23.20	45.00
Random-30-30-6	0.678	21.20	0.02	21.60	1.89	0.00	21.80	2.83	0.00	22.20	4.72	0.00	24.80	16.98	0.00	26.60	25.47
Random-30-30-8	0.864	25.20	0.03	25.20	0.00	0.00	25.40	0.79	0.00	25.40	0.79	0.00	28.40	12.70	0.00	29.00	15.08
Random-30-30-10	0.957	27.00	0.04	27.00	0.00	0.00	27.00	0.00	0.00	27.00	0.00	0.00	29.60	9.63	0.00	29.80	10.37
Random-40-40-2	0.156	11.00	0.02	11.60	5.45	0.00	15.00	36.36	0.00	13.00	18.18	0.00	16.00	45.45	0.00	22.20	101.82
Random-40-40-4	0.352	20.20	0.03	20.80	2.97	0.00	21.20	4.95	0.00	21.40	5.94	0.00	25.80	27.72	0.00	32.20	59.41
Random-40-40-6	0.582	28.20	0.05	28.80	2.13	0.00	28.80	2.13	0.00	29.00	2.84	0.00	33.20	17.73	0.00	35.60	26.24
Random-40-40-8	0.788	32.60	0.09	33.20	1.84	0.00	33.40	2.45	0.00	33.60	3.07	0.00	37.40	14.72	0.00	38.20	17.18
Random-40-40-10	0.902	35.40	0.11	35.40	0.00	0.00	35.60	0.56	0.00	35.80	1.13	0.00	39.20	10.73	0.00	39.00	10.17
Random-50-50-2	0.131	13.20	0.03	14.60	10.61	0.00	15.20	15.15	0.00	15.20	15.15	0.00	19.80	50.00	0.00	28.80	118.18
Random-50-50-4	0.301	23.80	0.07	25.00	5.04	0.00	26.00	9.24	0.00	26.00	9.24	0.00	30.80	29.41	0.00	38.80	63.03
Random-50-50-6	0.544	35.20	0.13	35.80	1.70	0.00	36.20	2.84	0.00	35.80	1.70	0.00	43.00	22.16	0.00	45.40	28.98
Random-50-50-8	0.746	40.40	0.20	40.40	0.00	0.00	41.60	2.97	0.00	41.80	3.47	0.00	46.80	15.84	0.00	46.80	15.84
Random-50-50-10	0.882	44.40	0.25	44.60	0.45	0.00	45.00	1.35	0.00	44.80	0.90	0.00	49.20	10.81	0.00	48.00	8.11
Random-50-100-2	0.189	21.20	0.13	21.80	2.83	0.00	23.00	8.49	0.00	22.60	6.60	0.00	29.40	38.68	0.00	28.80	35.85
Random-50-100-4	0.493	34.60	0.33	35.80	3.47	0.00	36.60	5.78	0.00	36.20	4.62	0.00	42.40	22.54	0.00	36.00	4.05
Random-50-100-6	0.783	42.40	0.56	43.20	1.89	0.00	43.20	1.89	0.00	43.40	2.36	0.00	49.40	16.51	0.00	43.60	2.83
Random-50-100-8	0.932	46.20	0.64	46.40	0.43	0.00	46.40	0.43	0.00	46.40	0.43	0.00	50.00	8.23	0.00	46.40	0.43
Random-50-100-10	0.985	47.80	0.69	47.80	0.00	0.00	47.80	0.00	0.00	47.80	0.00	0.00	50.00	4.60	0.00	47.60 *	-0.42
Random-75-75-2	0.081	15.80	0.10	17.40	10.13	0.00	22.00	39.24	0.00	19.20	21.52	0.00	25.60	62.03	0.00	39.80	151.90
Random-75-75-4	0.208	34.20	0.23	35.20	2.92	0.00	37.40	9.36	0.00	37.60	9.94	0.00	43.60	27.49	0.00	55.00	60.82
Random-75-75-6	0.402	50.00	0.48	50.60	1.20	0.00	53.00	6.00	0.00	52.40	4.80	0.00	60.40	20.80	0.00	64.00	28.00
Random-75-75-8	0.602	59.40	0.78	60.40	1.68	0.00	60.20	1.35	0.00	60.40	1.68	0.00	70.60	18.86	0.00	70.00	17.85
Random-75-75-10	0.754	64.80	1.01	65.40	0.93	0.00	66.00	1.85	0.00	65.80	1.54	0.00	72.80	12.35	0.00	71.60	10.49
Random-100-50-2	0.055	11.60	0.06	12.80	10.34	0.00	20.60	77.59	0.00	15.40	32.76	0.00	20.80	79.31	0.00	42.80	268.97
Random-100-50-4	0.102	26.40	0.11	27.60	4.55	0.00	35.20	33.33	0.00	32.20	21.97	0.00	35.20	33.33	0.00	43.20	63.64
Random-100-50-6	0.192	44.00	0.20	45.20	2.73	0.00	50.00	13.64	0.01	48.60	10.45	0.00	56.20	27.73	0.00	44.40	0.91
Random-100-50-8	0.292	56.20	0.30	57.80	2.85	0.00	61.60	9.61	0.01	61.00	8.54	0.00	69.20	23.13	0.00	46.40 *	-17.44
Random-100-50-10	0.406	67.20	0.43	68.00	1.19	0.00	71.20	5.95	0.01	70.80	5.36	0.00	79.40	18.15	0.00	47.00 *	-30.06
Random-100-100-2	0.061	20.20	0.23	22.00	8.91	0.00	27.20	34.65	0.00	24.00	18.81	0.00	33.00	63.37	0.00	51.40	154.46
Random-100-100-4	0.164	45.00	0.53	47.60	5.78	0.00	51.20	13.78	0.01	48.80	8.44	0.00	61.00	35.56	0.00	69.40	54.22
Random-100-100-6	0.317	64.60	1.09	65.60	1.55	0.00	66.20	2.48	0.01	67.40	4.33	0.00	81.80	26.63	0.00	85.80	32.82
Random-100-100-8	0.484	76.60	1.78	78.00	1.83	0.00	78.80	2.87	0.01	78.60	2.61	0.00	93.60	22.19	0.00	91.80	19.84
Random-100-100-10	0.643	84.60	2.45	85.60	1.18	0.00	85.80	1.42	0.01	86.00	1.65	0.00	97.00	14.66	0.00	95.60	13.00
Random-125-125-2	0.046	22.80	0.50	25.40	11.40	0.01	33.60	47.37	0.01	27.00	18.42	0.00	41.60	82.46	0.00	66.20	190.35
Random-125-125-4	0.127	53.00	1.16	55.40	4.53	0.01	60.20	13.58	0.01	58.20	9.81	0.00	73.00	37.74	0.00	91.40	72.45
Random-125-125-6	0.257	78.40	2.55	80.80	3.06	0.01	84.00	7.14	0.01	82.20	4.85	0.00	96.40	22.96	0.00	103.40	31.89
Random-125-125-8	0.403	93.60	4.36	94.80	1.28	0.01	98.20	4.91	0.01	96.80	3.42	0.00	110.60	18.16	0.00	114.00	21.79
Random-125-125-10	0.555	103.20	6.43	104.40	1.16	0.01	106.00	2.71	0.01	105.40	2.13	0.00	120.40	16.67	0.00	118.60	14.92
	**Total**	1646.20	28.22	1683.80	2.28	0.05	1765.80	7.27	0.16	1731.20	5.16	0.00	2020.00	22.71	0.00	1975.80	20.02

Carvalho & Soma																		
Dataset	Densidade	OPT	HBF2r			MCNh			PieceRank			Yuen3			Ashikaga & Soma			
			Time	Solution	%Gap	Time	Solution	%Gap	Time	Solution	%Gap	Time	Solution	%Gap	Time	Solution	%Gap	
Random-150-150-2	0.037	25.90	0.96	28.30	9.27	0.01	39.30	51.74	0.00	31.90	23.17	0.00	48.00	85.33	0.00	75.50	191.51	
Random-150-150-4	0.103	61.60	2.23	64.20	4.22	0.01	69.30	12.50	0.00	68.80	11.69	0.00	85.00	37.99	0.00	102.70	66.72	
Random-150-150-6	0.215	93.20	4.99	95.60	2.58	0.01	98.70	5.90	0.01	98.60	5.79	0.00	116.30	24.79	0.00	127.40	36.70	
Random-150-150-8	0.345	111.70	9.00	113.30	1.43	0.01	115.40	3.31	0.01	116.10	3.94	0.00	136.50	22.20	0.00	137.70	23.28	
Random-150-150-10	0.484	123.90	14.56	124.80	0.73	0.01	127.30	2.74	0.01	126.60	2.18	0.00	145.40	17.35	0.00	142.40	14.93	
Random-175-175-2	0.034	30.30	1.85	33.60	10.89	0.01	44.50	46.86	0.00	37.80	24.75	0.00	53.20	75.58	0.00	90.60	199.01	
Random-175-175-4	0.092	73.70	4.38	76.90	4.34	0.01	83.00	12.62	0.01	82.30	11.67	0.00	101.00	37.04	0.00	123.10	67.03	
Random-175-175-6	0.190	107.70	9.58	110.60	2.69	0.01	115.50	7.24	0.01	114.70	6.50	0.00	137.80	27.95	0.00	146.60	36.12	
Random-175-175-8	0.300	128.30	16.62	130.10	1.40	0.01	133.60	4.13	0.02	133.60	4.13	0.00	155.50	21.20	0.00	159.60	24.40	
Random-175-175-10	0.443	143.50	27.42	144.60	0.77	0.01	147.10	2.51	0.02	147.60	2.86	0.00	168.80	17.63	0.00	164.50	14.63	
Random-200-200-2	0.030	36.00	3.21	38.90	8.06	0.02	50.10	39.17	0.01	44.70	24.17	0.00	62.90	74.72	0.00	100.90	180.28	
Random-200-200-4	0.080	84.30	7.08	85.70	1.66	0.02	94.00	11.51	0.01	91.80	8.90	0.00	116.10	37.72	0.00	140.90	67.14	
Random-200-200-6	0.162	121.50	15.06	123.70	1.81	0.02	128.40	5.68	0.02	129.00	6.17	0.00	154.10	26.83	0.00	166.30	36.87	
Random-200-200-8	0.274	147.10	28.38	149.20	1.43	0.02	153.40	4.28	0.02	153.20	4.15	0.00	179.10	21.75	0.00	179.70	22.16	
Random-200-200-10	0.396	162.80	46.15	164.00	0.74	0.02	167.20	2.70	0.02	167.60	2.95	0.00	190.90	17.26	0.00	188.90	16.03	
	**Total**	1451.50	191.47	1483.50	2.20	0.20	1566.80	7.94	0.17	1544.30	6.39	0.00	1850.60	27.50	0.00	2046.80	41.01	

Frinhani, Carvalho & Soma																
Dataset	Densidade	HBF2r		MCNh			PieceRank			Yuen3			Ashikaga & Soma		
		Time	Solution	Time	Solution	%Gap	Time	Solution	%Gap	Time	Solution	%Gap	Time	Solution	%Gap
Random-400-400-2	0.015	51.27	74.20	0.17	99.70	34.37	0.03	82.60	11.32	0.00	124.70	68.06	0.00	199.5	168.87
Random-400-400-4	0.042	108.56	164.60	0.17	181.90	10.51	0.04	177.60	7.90	0.00	224.60	36.45	0.00	277.7	68.71
Random-400-400-6	0.088	229.28	242.50	0.16	254.80	5.07	0.05	254.80	5.07	0.00	306.60	26.43	0.00	332.2	36.99
Random-400-400-8	0.151	405.28	294.30	0.16	303.10	2.99	0.06	302.80	2.89	0.00	358.10	21.68	0.00	361.3	22.77
Random-400-400-10	0.224	631.77	323.70	0.16	330.10	1.98	0.07	331.40	2.38	0.00	384.00	18.63	0.00	375.1	15.88
Random-400-400-14	0.391	1263.60	357.90	0.16	361.40	0.98	0.09	361.70	1.06	0.00	397.50	11.06	0.00	385.7	7.77
Random-400-400-18	0.558	2135.36	374.30	0.16	376.30	0.53	0.10	375.60	0.35	0.00	399.80	6.81	0.00	391.5	4.60
Random-400-400-20	0.635	2667.68	379.00	0.16	380.30	0.34	0.10	380.40	0.37	0.00	400.00	5.54	0.00	393.7	3.88
Random-400-400-24	0.765	3943.05	386.10	0.16	386.70	0.16	0.11	386.70	0.16	0.00	400.00	3.60	0.00	396	2.56
Random-400-400-28	0.861	5486.73	390.00	0.16	390.20	0.05	0.13	390.10	0.03	0.00	400.00	2.56	0.00	397.5	1.92
Random-400-400-30	0.897	6351.33	391.80	0.16	391.60	-0.05	0.14	391.70	-0.03	0.00	400.00	2.09	0.00	397.7	1.51
Random-400-400-34	0.945	8233.64	393.90	0.17	393.80	-0.03	0.16	393.90	0.00	0.00	400.00	1.55	0.00	398.5	1.17
Random-600-600-2	0.010	276.39	106.60	0.57	145.80	36.77	0.05	125.40	17.64	0.01	187.10	75.52	0.00	306.7	187.71
Random-600-600-4	0.028	586.04	243.10	0.57	270.30	11.19	0.09	266.40	9.58	0.01	332.90	36.94	0.00	411.1	69.11
Random-600-600-6	0.059	1206.98	362.20	0.56	382.40	5.58	0.12	379.20	4.69	0.01	462.70	27.75	0.00	495.8	36.89
Random-600-600-8	0.103	2061.88	437.60	0.54	453.00	3.52	0.14	452.60	3.43	0.01	539.10	23.19	0.00	536.6	22.62
Random-600-600-10	0.156	3203.93	484.20	0.53	494.60	2.15	0.15	494.50	2.13	0.01	575.80	18.92	0.00	560.5	15.76
Random-600-600-14	0.278	6104.34	532.70	0.53	538.60	1.11	0.17	540.10	1.39	0.01	595.90	11.86	0.00	579.6	8.80
Random-600-600-18	0.417	10096.43	558.10	0.52	562.10	0.72	0.19	562.00	0.70	0.01	599.40	7.40	0.00	587.7	5.30
Random-600-600-20	0.487	12706.91	566.30	0.52	569.40	0.55	0.20	569.90	0.64	0.01	599.90	5.93	0.00	590	4.19
Random-600-600-24	0.616	18712.84	576.80	0.53	577.30	0.09	0.23	577.30	0.09	0.01	600.00	4.02	0.00	592.5	2.72
Random-600-600-28	0.727	25967.73	583.60	0.53	584.80	0.21	0.26	584.20	0.10	0.01	600.00	2.81	0.00	594.3	1.83
Random-600-600-30	0.778	30155.83	585.90	0.53	586.10	0.03	0.27	586.60	0.12	0.01	600.00	2.41	0.00	595.9	1.71
Random-600-600-34	0.855	39612.55	589.70	0.54	590.30	0.10	0.30	590.00	0.05	0.01	600.00	1.75	0.00	597	1.24
Random-600-600-38	0.909	56468.43	592.80	0.54	591.80	-0.17	0.34	591.60	-0.20	0.01	600.00	1.21	0.00	597.8	0.84
Random-600-600-40	0.931	50018.02	592.20	0.55	592.70	0.08	0.36	592.90	0.12	0.01	600.00	1.32	0.00	597.8	0.95
	**Total**	288685.87	10584.10	9.51	10789.10	1.94	3.96	10742.00	1.49	0.14	11688.10	10.43	0.00	11949.70	12.90

Frinhani, Carvalho & Soma													
Dataset	Densidade	MCNh		PieceRank			Yuen3			Ashikaga & Soma			
		Time	Solution	Time	Solution	%Gap	Time	Solution	%Gap	Time	Solution	%Gap	
Random-800-800-2	0.009	1.49	190.40	0.09	161.50	-15.18	0.01	244.30	28.31	0.00	397.50	108.77	
Random-800-800-4	0.021	1.47	364.00	0.15	354.70	-2.55	0.01	448.20	23.13	0.00	553.00	51.92	
Random-800-800-6	0.044	1.45	506.30	0.20	504.20	-0.41	0.01	619.10	22.28	0.00	660.10	30.38	
Random-800-800-8	0.077	1.42	599.60	0.22	596.60	-0.50	0.01	717.40	19.65	0.00	721.20	20.28	
Random-800-800-10	0.117	1.38	658.40	0.25	658.50	0.02	0.01	768.00	16.65	0.01	746.80	13.43	
Random-800-800-14	0.216	1.36	720.30	0.28	720.50	0.03	0.01	794.20	10.26	0.00	771.50	7.11	
Random-800-800-18	0.336	1.34	748.70	0.32	749.80	0.15	0.01	799.30	6.76	0.00	783.30	4.62	
Random-800-800-20	0.394	1.34	757.80	0.34	758.40	0.08	0.01	799.70	5.53	0.00	786.10	3.73	
Random-800-800-24	0.514	1.35	770.50	0.37	770.60	0.01	0.01	799.90	3.82	0.00	790.40	2.58	
Random-800-800-28	0.626	1.35	778.40	0.42	778.00	-0.05	0.01	800.00	2.77	0.00	792.60	1.82	
Random-800-800-30	0.678	1.36	781.10	0.44	781.20	0.01	0.01	800.00	2.42	0.00	794.00	1.65	
Random-800-800-34	0.767	1.52	785.30	0.49	785.30	0.00	0.01	800.00	1.87	0.00	795.50	1.30	
Random-800-800-38	0.836	1.37	788.80	0.53	788.90	0.01	0.02	800.00	1.42	0.00	796.30	0.95	
Random-800-800-40	0.864	1.37	789.90	0.57	789.80	-0.01	0.02	800.00	1.28	0.00	796.60	0.85	
Random-800-800-44	0.911	1.41	792.10	0.62	791.90	-0.03	0.02	800.00	1.00	0.00	797.80	0.72	
Random-800-800-48	0.944	1.39	793.11	0.69	793.20	0.01	0.02	800.00	0.87	0.00	797.67	0.57	
Random-800-800-50	0.957	1.37	794.18	0.74	793.80	-0.05	0.02	800.00	0.73	0.00	798.09	0.49	
Random-1000-1000-2	0.007	3.37	237.30	0.13	200.80	-15.38	0.03	302.00	27.27	0.01	488.90	106.03	
Random-1000-1000-4	0.017	3.31	449.40	0.22	439.30	-2.25	0.03	549.40	22.25	0.01	685.90	52.63	
Random-1000-1000-6	0.036	3.40	635.80	0.29	630.90	-0.77	0.03	775.90	22.04	0.01	827.80	30.20	
Random-1000-1000-8	0.063	3.23	748.20	0.34	748.60	0.05	0.03	903.00	20.69	0.01	896.10	19.77	
Random-1000-1000-10	0.095	3.18	822.30	0.37	821.20	-0.13	0.03	961.70	16.95	0.01	932.80	13.44	
Random-1000-1000-14	0.178	3.12	899.20	0.42	899.10	-0.01	0.03	992.50	10.38	0.01	964.60	7.27	
Random-1000-1000-18	0.277	3.11	936.00	0.48	935.10	-0.10	0.03	999.00	6.73	0.01	979.30	4.63	
Random-1000-1000-20	0.329	3.13	945.80	0.50	945.90	0.01	0.03	999.40	5.67	0.01	981.50	3.77	
Random-1000-1000-24	0.438	3.14	962.50	0.55	962.90	0.04	0.03	1000.00	3.90	0.01	988.00	2.65	
Random-1000-1000-28	0.544	3.60	972.40	0.62	972.10	-0.03	0.03	1000.00	2.84	0.01	991.10	1.92	
Random-1000-1000-30	0.594	3.50	976.10	0.65	975.30	-0.08	0.03	1000.00	2.45	0.01	992.20	1.65	
Random-1000-1000-34	0.685	3.30	981.80	0.71	981.60	-0.02	0.03	1000.00	1.85	0.01	993.40	1.18	
Random-1000-1000-38	0.765	3.28	985.10	0.77	985.00	-0.01	0.03	1000.00	1.51	0.01	994.50	0.95	
Random-1000-1000-40	0.797	3.24	986.90	0.80	986.70	-0.02	0.03	1000.00	1.33	0.01	995.80	0.90	
Random-1000-1000-44	0.855	3.29	989.30	0.88	989.20	-0.01	0.03	1000.00	1.08	0.01	996.30	0.71	
Random-1000-1000-48	0.901	3.22	991.40	0.98	991.40	0.00	0.03	1000.00	0.87	0.01	997.00	0.56	
Random-1000-1000-50	0.918	3.26	991.90	1.04	991.60	-0.03	0.03	1000.00	0.82	0.01	997.40	0.55	
Random-1000-1000-54	0.946	3.21	993.30	1.14	992.90	-0.04	0.03	1000.00	0.67	0.01	997.60	0.43	
	Total	82.63	27123.59	17.59	27026.50	-0.36	0.76	28873.00	6.45	0.19	29278.66	7.95	

METHODS REFERENCES

**Yuen3**	Yuen BJ. Improved heuristics for sequencing cutting patterns, European Journal of Operational Research, Elsevier, v. 87, n. 1, p. 57-64, 1995.
**Ashikaga & Soma**	Ashikaga FM, Soma NY. A heuristic for the minimization of open stacks problem, Pesquisa Operacional, v. 29, p. 439-450, 2009.
**Chu & Stuckey**	Chu G, Stuckey PJ. Minimizing the maximum number of open stacks by customer search, Inter. Conf. on Principles and Practice of Constraint Prog., Springer, p. 242-257, 2009.
**HBF2r**	Carvalho MAM, Soma NY. A breadth-first search applied to the minimization of the open stacks, Jour. of Operational Research Society, Springer, v. 66, n. 6, p. 936-946, 2015.
**MCNh**	Becceneri JC, Yanasse HH, Soma NY. A method for solving the minimization of the maximum number of open stacks problem within a cutting process, Computers & Operations Research, Elsevier, v. 31, n. 14, p. 2315-2332, 2004.

DATASETS REFERENCES

**First Constraint Modelling Challenge**	Smith BM, Gent IP. Constraint modelling challenge 2005, IJCAI 2005 Fifth Workshop on Modelling and Solving Problems with Constraints, p. 1-8, 2005.
**SCOOP Project**	SCOOP Project - Sheet Cutting and Process Optimization for furniture enterprises, www.scoop-project.net, Visited in: 28/03/2018.
**Faggioli & Bentvoglio**	Faggioli E, Bentivoglio CA. Heuristic and exact methods for the cutting sequencing problem, European Journal of Operational Research, Elsevier, v. 110, n. 3, p. 564-575, 1998.
**Chu & Stuckey**	Chu G, Stuckey PJ. Minimizing the maximum number of open stacks by customer search, Inter. Conf. on Principles and Practice of Constraint Prog., Springer, p. 242-257, 2009.
**Carvalho & Soma**	Carvalho MAM, Soma NY. A breadth-first search applied to the minimization of the open stacks, Jour. of Operational Research Society, Springer, v. 66, n. 6, p. 936-946, 2015.
