# Supplementary material for: A PageRank-based heuristic for the minimization of open stacks problem
Source: PLoS One. 2018 Aug 30;13(8):e0203076. doi: 10.1371/journal.pone.0203076 (PMC6117050; doi:10.1371/journal.pone.0203076)
Supplement: S1 Dataset — Contains the files and links to get the datasets used in the experiments. (ZIP) [file pone.0203076.s002.zip › MOSP_Instances/S1. Dataset - README FIRST.pdf]

PLOS ONE - Supporting Information

S1. Dataset – MOSP Instances Files

Frinhani, Carvalho & Soma - A PageRank-based heuristic for the minimization of open stacks problem
July, 2018

In this Supporting Information each folder contains the instance files of the following datasets:

- First Constraint Modeling Challenge
- Faggioli & Bentivoglio
- SCOOP Project
- Chu & Stuckey

All matrices of these files are pattern x piece. In each folder the "Dataset_Description" file contains details about the respective dataset and file format.

The files related to Carvalho & Soma dataset, DOI 10.13140/2.1.3035.4886, are available at the Research Gate public repository. The matrices are piece x pattern:

https://www.researchgate.net/profile/Marco_Carvalho7/publication/267864061_Minimization_of_Open_Stacks_Problem_MOSP_or_Minimization_of_Open_Orders_Problem_MOOP_Instances/data/545b64cd0cf2f1dbcbc9e11c/mosp-instances.zip

----

The files related to Frinhani, Carvalho & Soma datasets, DOI 10.13140/RG.2.2.31716.48006/1, are available at the Research Gate public repository. The matrices are pattern x piece:

https://www.researchgate.net/profile/Rafael_De_Magalhaes_Dias_Frinhani/publication/324497787_Large_datasets_for_the_MOSP/data/5b6c9dda299bf14c6d97d073/MOSP-FrinhaniCarvalhoSoma.zip
